# Cap-independent translation and a precisely located RNA sequence enable SARS-CoV-2 to control host translation and escape anti-viral response

**DOI:** 10.1093/nar/gkac615

**Published:** 2022-07-18

**Authors:** Boris Slobodin, Urmila Sehrawat, Anastasia Lev, Daniel Hayat, Binyamin Zuckerman, Davide Fraticelli, Ariel Ogran, Amir Ben-Shmuel, Elad Bar-David, Haim Levy, Igor Ulitsky, Rivka Dikstein

**Affiliations:** Department of Biomolecular Sciences, The Weizmann Institute of Science, Rehovot 76100, Israel; Department of Biomolecular Sciences, The Weizmann Institute of Science, Rehovot 76100, Israel; Department of Cancer Biology and Genetics, Memorial Sloan Kettering Cancer Center, New York, NY 10065, USA; Department of Biomolecular Sciences, The Weizmann Institute of Science, Rehovot 76100, Israel; Department of Biomolecular Sciences, The Weizmann Institute of Science, Rehovot 76100, Israel; Department of Biological Regulation, The Weizmann Institute of Science, Rehovot 76100, Israel; Gladstone/UCSF Center for Cell Circuitry, Gladstone Institutes, San Francisco, CA 94158, USA; Department of Biomolecular Sciences, The Weizmann Institute of Science, Rehovot 76100, Israel; Department of Biomolecular Sciences, The Weizmann Institute of Science, Rehovot 76100, Israel; Department of Infectious Diseases, Israel Institute for Biological Research, Ness-Ziona 7410001 Israel; Department of Infectious Diseases, Israel Institute for Biological Research, Ness-Ziona 7410001 Israel; Department of Infectious Diseases, Israel Institute for Biological Research, Ness-Ziona 7410001 Israel; Department of Biological Regulation, The Weizmann Institute of Science, Rehovot 76100, Israel; Department of Biomolecular Sciences, The Weizmann Institute of Science, Rehovot 76100, Israel

## Abstract

Translation of SARS-CoV-2-encoded mRNAs by the host ribosomes is essential for its propagation. Following infection, the early expressed viral protein NSP1 binds the ribosome, represses translation, and induces mRNA degradation, while the host elicits an anti-viral response. The mechanisms enabling viral mRNAs to escape this multifaceted repression remain obscure. Here we show that expression of NSP1 leads to destabilization of multi-exon cellular mRNAs, while intron-less transcripts, such as viral mRNAs and anti-viral interferon genes, remain relatively stable. We identified a conserved and precisely located cap-proximal RNA element devoid of guanosines that confers resistance to NSP1-mediated translation inhibition. Importantly, the primary sequence rather than the secondary structure is critical for protection. We further show that the genomic 5′UTR of SARS-CoV-2 drives cap-independent translation and promotes expression of NSP1 in an eIF4E-independent and Torin1-resistant manner. Upon expression, NSP1 further enhances cap-independent translation. However, the sub-genomic 5′UTRs are highly sensitive to eIF4E availability, rendering viral propagation partially sensitive to Torin1. We conclude that the combined NSP1-mediated degradation of spliced mRNAs and translation inhibition of single-exon genes, along with the unique features present in the viral 5′UTRs, ensure robust expression of viral mRNAs. These features can be exploited as potential therapeutic targets.

## INTRODUCTION

Most viruses do not encode functional translation machinery and rely on the host cell to translate their genetic information into proteins. This makes viruses obligatory parasites that exploit the host translation apparatus for successful proliferation. Such an absolute dependence turns the translational regulation into the Achilles heel of viral infection and propagation. Infected cells attenuate cap-dependent translation to prevent the translation of viral proteins (1). Translation that does not rely on the 5′cap structure, such as via internal ribosome entry sites (IRESs), is a major strategy employed by multiple positive single-stranded RNA viruses to support the expression of viral mRNAs when the cap-dependent translation is inhibited. IRES activity enables eIF4E-independent translation and, therefore, is particularly important upon inhibition of mTOR complex (2). In parallel, multiple viruses attempt to enforce mTOR activation to relieve the translational repression (3). Notably, the cap-dependent and independent translation initiation pathways are not mutually exclusive and may cooperate to enhance viral-specific translational yield, as was shown for HCV (4).

SARS-CoV-2 is a betacoronavirus encoding a long 5′-capped and polyadenylated positive single-stranded RNA genome that serves as an immediate mRNA template for the translation of ORF1ab, two precursor polyproteins that are proteolytically cleaved to form 16 non-structural proteins (5). In addition, the SARS-CoV-2 genome gives rise to multiple sub-genomic mRNAs that are synthesized via discontinuous RNA-templated transcription and, similarly to the genomic RNA, bear 5′caps and poly(A) tails (6). The presence of the 5′cap on all viral mRNAs stresses the importance of cap-dependent translation initiation for SARS-CoV-2 and raises questions regarding the ability of the virus to oppose the cellular attenuation of cap-dependent translation.

NSP1, the non-structural protein 1, is a major virulence factor encoded by SARS-CoV-2. NSP1 directly interacts with host ribosomes via its C’-terminal moiety (7,8), repressing translation and inducing mRNA degradation in infected cells (9–11). Particularly, NSP1 was shown to associate with 40S ribosomal subunits at the mRNA entry channel, hindering the access of mRNAs to the ribosome. Interestingly, translation of innate immune genes is particularly repressed in the infected cells (10), enabling the virus to evade the host's innate defense. Viral 5′UTRs were shown to protect mRNAs from NSP1-mediated inhibition (10,12), and although the stem–loop element 1 (SL1) within viral 5′UTRs was suggested to play a role (13), the exact motif remains insufficiently characterized.

In this study, we show that NSP1 promotes degradation of host multi-exon mRNAs, while exerting a milder effect on the stability of intron-less transcripts. Using single-exon reporter mRNAs, we demonstrate that these transcripts are repressed by NSP1 primarily at the translation level. We identified a specific RNA sequence within the SARS-CoV-2 genomic and sub-genomic 5′UTRs that enables viral mRNAs to escape repression. This element depends on its nucleotide composition rather than the secondary structure and is characterized by the absence of guanosines and its precise location relative to the 5′end, two features that are conserved in several coronaviruses. We show that this element is sufficient for robust heterologous expression of DNA-encoded genes in NSP1-expressing cells. We also found that the genomic version of the viral 5′UTR that precedes NSP1 can promote cap-independent translation, supporting eIF4E-independent, Torin1-resistant initiation. These features enable the highly efficient expression of NSP1 even upon prolonged arrest of cap-dependent translation. In contrast, the sub-genomic 5′UTR exhibits high sensitivity to eIF4E availability. These findings unravel the molecular strategies employed by SARS-CoV-2 to hijack and adjust the host translation machinery for viral propagation and to overcome both inhibition of cap-dependent translation and induction of interferons.

## MATERIALS AND METHODS

### Reagents

#### DNA constructs

To create HA-tagged NSP1 lacking viral 5′UTR for expression in cell culture (pCRUZ-HA-NSP1), NSP1 was obtained from the Forchheimer plasmid bank (WIS, Israel) and cloned into pCRUZ-HA plasmid (sc-5045, Santa Cruz Biotechnology) using restriction-free cloning and primers #1, 2 (here and on, see Table S1 for primers’ sequences). To create His-tagged NSP1 lacking viral 5′UTR for bacterial expression, NSP1 was PCR amplified using primers #3,4 and inserted into pET28 plasmid with 14xHis-bdSumo tag (14). The insertion of NSP1 removed the bdSumo but maintained the 14xHis tag. To create HA-tagged NSP1 with the viral 5′UTR for expression in cell culture (pcDNA3.1-TSS-5′UTR-HA-NSP1), HA-NSP1 was PCR-amplified from the pCRUZ-HA-NSP1 plasmid using primers #5,6 and introduced immediately after the transcription start site of the pcDNA3.1(–) plasmid using restriction-free cloning. This plasmid (pcDNA3.1-TSS-5′UTR-HA-NSP1) was used in this study to express HA-NSP1 in mammalian cultured cells, unless indicated otherwise. The genomic 5′UTR of SARS-CoV-2 was created by sequential annealing and amplification of primers #13–17. The combined 5′UTR was introduced between KpnI and BamHI sites of pcDNA5/FRT/TO plasmid (ThermoFisher Scientific); Renilla (Rluc) reporter gene was introduced into the same plasmid using BamHI-NotI sites. For bi-cistronic plasmids, Firefly (Ffly) reporter gene bearing Hisx6 tag was amplified using primers #18,19 and cloned between HindIII-KpnI sites of pcDNA5/FRT/TO plasmid. This plasmid was used to both express Firefly gene in cells and synthesize Firefly mRNA *in vitro* using T7 promoter. Rluc was amplified using primers #20,21 and introduced using XhoI site of the same plasmid to create a bi-cistronic expression vector where both reporters have distinct translation start and stop codons. After cloning, the XhoI site was preserved only before the Rluc gene and used for insertion of tested DNA sequences in both orientations. For this purpose, the SARS-CoV-2 genomic 5′UTR was amplified using primers #22,23; EMCV IRES was amplified from the TRex-IRES-RLUC plasmid (15) with #24,25 (originally, this IRES was amplified from the pIRES2-EGFP plasmid (Clontech). Other IRES sequences were amplified from plasmids kindly provided by M. Lopez-Lastra (Universidad Católica de Chile) (16) and cloned between the KpnI and BamHI sites of TRex-Rluc plasmid. To create a plasmid with sub-genomic 5′UTR fused to Rluc, the Rluc gene was first amplified with primers #26,27 and the resulting product was further re-amplified with primers #27,28. The resulting product was used to run a PCR using pcDNA5/FRT/TO plasmid as a template. The resulting plasmid had 16nt between the transcription start site (TSS) and the beginning of the viral 5′UTR. To create plasmid bearing 155nt between the TSS and the viral 5′UTR, the cassette including the sub-genomic 5′UTR and Rluc gene was amplified using primers #13,29 and inserted between KpnI-NotI sites of pcDNA5/FRT/TO. The same strategy was used to create plasmid bearing the genomic 5′UTR. To create a plasmid with viral 5′UTR placed immediately after the TSS, primers # 13,30 were used to amplify the 5′UTR-Rluc cassette and the product was re-amplified using primers #31,32. The resulting product was used in a PCR to amplify pcDNA3.1(–) plasmid as template. All constructed plasmids were subjected to Sanger sequencing.

#### Restriction-free cloning

For restriction-free cloning, a PCR product with ends overlapping the destination plasmid was used for a subsequent PCR using the destination plasmid as a template. The resulting product was cleaned, concentrated, treated with DpnI enzyme for 1–2 h, and transformed into bacteria. Grown colonies were screened using colony PCR using HyTaq mix (HyLabs, Israel).

#### Plasmid DNA preparation

Plasmid DNA of interest was transformed into competent DH5 alpha bacteria using the heat shock method and plated on LB plates supplemented with either Ampicillin (200 μg/ml) or Kanamycin (50 μg/ml). Grown colonies were isolated, grown overnight in liquid LB medium supplemented with the relevant antibiotic, and plasmid DNA was extracted using either miniprep or maxiprep kits.

#### In vitro RNA synthesis

DNA templates bearing T7 promoter were produced by PCR amplification (Kappa HiFi Hotstart, Roche) of plasmids encoding for Renilla reporter using primers detailed in Table S1. Typically, primer #33 was used to add poly(A) tail of 30nt, while primers #34–47 introduced both the T7 promoter and the indicated sequence manipulations. PCR products were cleaned using columns (Qiagen) and used for *in-vitro* transcription reactions using RiboMAX kit (Promega) according to the manufacturer's instructions. The reactions were treated with DNAse (15 min at 37°C) and the remaining RNA was recovered using Direct-zol RNA mini prep kit (Zymo research) according to the protocol. For RNA capping we used the Vaccinia capping kit (NEB) followed by Direct-zol RNA mini prep kit (Zymo research).

#### RNA isolation and RT-qPCR assay

RNA was isolated from cultured cells using BIO TRI RNA reagent (Bio-Lab Chemicals) and Direct-zol RNA MiniPrep kit (Zymo research), as instructed by the manufacturers. When the cells were transfected with plasmids prior to RNA extraction, DNAse treatment (Turbo DNAse, Invitrogen) was performed, and the RNA samples were re-isolated. To test the efficacy of DNAse treatment, these samples were subjected to qPCR without prior reverse transcription. Following the isolation, RNA was typically reconstituted in 25 μl of sterile nuclease-free water (Bio-Lab Chemicals) and stored at –20°C. To assess RNA integrity, 5μl of the total RNA were resolved on agarose gels and visualized. Reverse transcription (RT) was done using High Capacity cDNA Reverse Transcription Kit (Applied Biosystem) using either random hexamers or Renilla-specific primer (Table S1, #79, 0.5 μM), according to the manual. Typically, 5 μl of total isolated RNA were taken for a single RT reaction and diluted 1:5 with water afterwards. Quantitative real time PCR (qPCR) experiments were performed in total reaction volume of 10 μl with qPCRBIO SyGreen Blue Mix Hi-ROX (PCR BIOSYSTEMS) reagent on 384-wells plates (Axygen) using Viia7 (Thermo Fisher Scientific) instrument in standard conditions. Typically, two or three technical repeats were done for each biological sample and their average values were taken for subsequent calculations done according to the ΔΔC_t_ method formula. In most cases, such as in the experiments presented in Supplementary Figure S3B, C), the tested RNA molecules encoding different 5′UTRs were mixed, used as a single pooled sample, and later de-convoluted according to the barcode present in their 3′end. All used primers (Table S1) targeted exons in their respective genes.

#### Nsp1 protein purification for in vitro assay

The pET28 plasmid encoding for His-tagged NSP1 was transformed into BL21(DE3) *E. coli* cells and a single colony was inoculated into 5 ml of LB supplemented with Kanamycin (50 μg/ml) and incubated with shaking at 37°C. After 8 h, the bacteria were transferred into Erlenmeyer flask with 1 l of LB supplemented with Kanamycin and grown to O.D ∼0.5 at 37°C. IPTG (0.5 mM) was added to the medium and incubated orbitally shaking at 16°C overnight. The bacteria were pelleted (4000 RPM, 20 min) and resuspended in 20 ml of resuspension buffer (500 mM NaCl, 30 mM HEPES, 5 mM MgCl). The resuspended bacteria were sonicated (probe sonicator, 12 cycles of 30 s on + 30 s off) and centrifuged to pellet and remove the cellular debris. Supernatant was then loaded on 500 ul nickel-NTA beads, washed 4 times with 10 ml wash buffer (resuspension buffer with 20 mM imidazole) and eluted with 5 ml resuspension buffer supplemented with 300 mM imidazole. The eluate was subjected to buffer exchange in a dialysis bag (10 kDa) against resuspension buffer without imidazole and used for *in vitro* assays. This protocol is based on previously reported procedure for recombinant NSP1 isolation (8).

#### In-vitro translation assay

*In vitro* translation in nuclease-treated Rabbit Reticulocyte Lysate System (RRL, Promega) was carried out as recommended by the manufacturer with slight modifications. The final volume of the reaction mixtures was 12.5 μl. The mixtures were preincubated for 10 min with either NSP1 or BSA protein (5 ng/μl). Indicated *in-vitro* transcribed and capped mRNAs were added to the RRL reactions (6.25 ng/μl) and incubated at 30°C for 1 h. Samples of 3 μl were then subjected to Renilla activity measurements.

#### mRNA library preparation for MARS-seq

Total RNA was isolated from HEK293T cells using BIO TRI RNA reagent (Bio-Lab Chemicals) and mRNA was captured using Oligo d(T)25 magnetic Beads (NEB) according to manufacturer's protocol. Poly(A)-purified RNAs were taken for library preparation using a derivation of MARS-seq (massively parallel RNA sequencing) as described (17). Briefly, 10 ng of poly(A)+ RNA was taken for the first reverse transcription reaction using Illumina barcoded RT1 primer. Resultant barcoded cDNA samples were subsequently pooled according to Ct values of a house-keeping gene (GAPDH) (Quality control 1). Pooled cDNA was treated with Exonuclease I (NEB) to remove excess primers followed by second strand synthesis. After that, *in-vitro* transcription was performed using T7 RNA Polymerase (NEB) to generate RNA, which was later fragmented and ligated to an adaptor consisting of RD2 using T4 RNA ligase I (NEB) followed by the second reverse transcription reaction. The library so formed was amplified using Kapa Hifi ready mix (Roche). The amplified RNA libraries were sequenced using a high-throughput 75 bp kit (Illumina FC404-2005) on NEXTseq 500 sequencer.

#### Polysomal isolation

Cultured cells (24 h after transfection, at ∼80% confluency) were treated with 100 μg/ml cycloheximide for 5 min and washed with cold polysome buffer (20 mM Tris pH 8, 140 mM KCl, 5 mM MgCl_2_ and 100 μg/ml cycloheximide). Cells were collected in 500-μl polysome buffer supplemented with 0.5% Triton, 0.5% Deoxycholic acid (Sigma), 1.5 mM DTT, 150 units RNase inhibitor and 5 μl protease inhibitor cocktail. After mechanical disruption, the samples were centrifuged at 12 000 rpm for 5 min at 4ºC. The cleared lysates were loaded onto sucrose density gradient (10–50%) and centrifuged at 38 000 rpm for 105 min at 4ºC. Gradients were fractionated with continuous absorbance 254 nm (A254) measurement using ISCO absorbance detector UA-6. Fractions were pooled according to their absorbance into free, light and heavy ribosomal fractions. A whole sum of absorbance (*A*_254_) for 80S peak was calculated and marked as a monosomal fraction. Similarly, the total sum of absorbance (*A*_254_) for both light and heavy fractions was calculated as a polysomal fraction. For protein extraction, polysomal fractions collected from sucrose gradients were treated with 0.25 volumes of ice-cold 100% Trichloroacetic acid and 0.05% sodium deoxycholate to precipitate total proteins for 30 min on ice. The samples were then centrifuged at 4°C 20 000 *g* for 30 min. The protein pellets were carefully washed twice with pure acetone, air dried and reconstituted in a 2× sample loading buffer. The protein samples were loaded into 12% SDS-PAGE gel and subjected to Western blot analysis.

#### Puromycin labelling

Cells were transfected with plasmids encoding either HA-NSP1 or eGFP and 24 h later, puromycin (10 μg/ml) was added for 5 min. The cells were then collected on ice, lysed using RIPA buffer (Sigma) and subjected to 10% SDS-PAGE followed by Western blotting.

#### Western blotting

Protein extracts were resolved on SDS-PAGE using standard equipment (Bio-Rad) and transferred to PVDF membranes in buffer containing 20% methanol. Quality of transfer was examined by Ponceau staining (0.1% ponceau in 20% acetic acid) and membranes were blocked in skim milk (5% w/v) solution. The membranes were probed with the indicated antibodies overnight at 4°C, washed in washing buffer and probed with secondary HRP-conjugated antibodies (Jackson Immunoresearch). Primary antibodies used: anti-puromycin (Millipore Cat#MABE343), anti-alpha-tubulin (Sigma Cat# T5168), anti-GFP (Abcam Cat#ab1218), anti-HA (Abcam Cat#ab9110), anti-RPS3A (Antobody Verify, Cat#AAS38561C). After treatment with ECL reagent (Azure Biosystems), images were captured using Licor Fc imaging system and the signal intensities were calculated using ImageStudio software.

#### Luminescence assay

The cells were lysed in reporter lysis buffer (Promega), according to the volumes recommended by the manufacturer. For Renilla substrate, Coelenterazine (CTZ) reagent (Bio Gold, St. Louis, USA) was dissolved in methanol to stock concentration of 2.5 mg/ml and equilibrated with 0.1N HCl. It was further diluted 1:1000 in phosphate buffer containing 80 mM K_2_HPO_4_ and 20 mM KH_2_PO_4_. Signals were detected in white 96-well plates using Modulus microplate luminometer reader (Turner Biosystems) combining 5 μl cell lysate and 50 μl of CTZ solution. For Firefly assay, 5 μl of cell lysates were combined with 25 μl of luciferin reagent (0.5 mM ATP, 33.3 mM DTT, 0.2 μg/μl co-enzyme A, 0.5 mM d-luciferin in reagent buffer [52% (w/v) of (MgCo_3_)_4_ Mg(OH)_2_·5H_2_O, 20 mM tricine, 5.34 mM MgSO_4_·7H_2_O, pH 7.8]). Where applicable, the relative signal ratio (e.g. Renilla/Firefly) was calculated and presented; see Table S2 for the raw data.

### Biological resources

#### Cultured cell lines

MCF7 and HEK293 cells were from ATCC; MRC5 and Vero E6 cells were generously provided by Zvi Livneh and Yosef Shaul, respectively (WIS, Israel). The cells were grown in DMEM media (Gibco) supplemented with 10% bovine serum and 1% penicillin/streptomycin (Gibco) in 5% CO_2_-buffered incubators at 37°C. Cells were split twice per week and kept in culture for up to 8 weeks.

#### Cell viability upon SARS-CoV-2 infection

SARS-CoV-2 (GISAID accession EPI_ISL_406862) was kindly provided by Bundeswehr Institute of Microbiology, Munich, Germany. Virus stocks were propagated (four passages) and titered on Vero E6 cells (Vero E6, ATCC^®^ CRL-1586™). Handling and working with SARS-CoV-2 virus were conducted in a BSL3 facility in accordance with the biosafety guidelines of the Israel Institute for Biological Research (IIBR). Vero E6 cells were seeded at a density of 3 × 10^4^ cells per well in 96-well plates. After overnight incubation, cells were treated in three replicates with Torin-1 (Fisher Scientific) or Remdesivir (Medchemexpress) as indicated. Cells were infected 1 h later with SARS-CoV-2 (MOI, 0.01–0.015). Cell viability was determined 72 h after infection by using the Cell Proliferation Kit (XTT based, Biological Industries, Israel) according to manufacturer's protocol. For positive control, cells were treated with Remdesivir, for negative control cells were not treated prior to infection.

#### Cell growth kinetics and proliferation assays

To visualize cellular growth, cells were seeded in six-well plates, treated as indicated, and grown for 48–72 h in optimal conditions. The cells were washed once in PBS, treated with PBS-formaldehyde (4%) for 20 min at room temperature and stained with crystal violet stain (Merck). After extensive washing, the plates were air-dried and documented. To monitor growth kinetics, the cells were seeded on 96-well plates, treated as indicated and subjected to CellTiter-Glo luminescent cell viability assay (Promega) according to the manufacturer's instructions.

#### Transfections

DNA plasmids were transfected using JetPrime reagent (Polyplus transfection). Typically, the medium was changed after 4 h, and cells were grown for 24–72 h prior to further analysis. Efficiencies of transfections were examined 20–24 h post-transfection using the GFP signal in eGFP-transfected cells. Typically, transfection efficiencies ranged between 70% and 80% in MCF7 and MRC5 cells, and 80–90% in 293 cells. siRNA transfections were done using Lipofectamine RNAiMax reagent (Thermo Fisher Scientific). The media were replaced 24 h after transfections and the cells were collected after additional 48 h. When indicated, cells were manipulated (e.g. transfected with DNA plasmids) within this time. *In-vitro* synthesized mRNAs were transfected using Lipofectamine 2000 reagent (Thermofisher scientific) according to the protocol provided by the manufacturer. Briefly, mRNA was mixed with Opti-MEM solution (Thermofisher scientific) and Lipofectamine reagent and incubated at room temperature for 10 min. The growing media were replaced with pre-warmed Opti-MEM solution and the transfection solution was added to cells. The transfection solution was replaced after 2 h to pre-warmed growth media and the cells were incubated at optimal conditions for 5 h prior to lysis and subsequent luminescence assay.

#### Computational resources

Raw data were processed using UTAP (18) with default parameters. Corrected counts were normalized by mouse Poly(A)+ enriched RNA, which was added as a spike to all samples and mapped to mouse genome using STAR (19) not allowing mismatches. Percent of uniquely mapped reads per sample was used as a normalization factor. Biological replicates were averaged and means were used for fitting a nonlinear least-squares model assuming first-order decay kinetics: $C\ = {C}_0\ {{\rm e}}^{ - {k}_{{\rm decay}}t}$, while corrected counts at *t* = 0 were used as *C*_0_ and inverse of standard errors of the corrected count means were used as weights for fitting. Half lives of all genes were then calculated using the following equation: *t*_1/2_ = ln(2)/*k*_decay_. Genes with mean corrected read count <5 at *t* = 0 were removed from subsequent analysis. Negative half life values and half lives >24 h were set to 24h.

#### Statistical analysis

Statistical significance was calculated using Student's *t*-tests with one-tailed distribution. Significance symbols in all experiments are: *P < 0.05; **P < 0.01; ***P < 0.001, ****P < 0.0001.

## Results

### NSP1 robustly destabilizes multi-exon mRNAs while moderately inhibiting translation initiation

**Figure 1.**
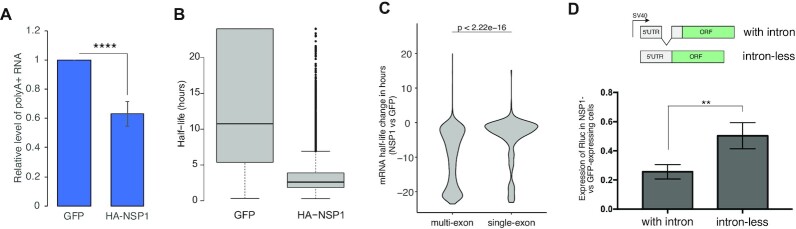
Expression of NSP1 destabilizes host mRNAs. (**A**) HEK293 cells were transfected with plasmids encoding either eGFP or HA-NSP1 (4 μg DNA per 10-cm dish) and collected after 24 h. Isolated RNA was subjected to poly(A)-dependent enrichment, quantified, and plotted in a relative manner; *n* = 5, the bar represents SE. (**B**) Cells transfected as detailed in (A) were treated with Actinomycin D (7.5μg/ml) for 0, 2, 4 and 6 h, harvested and subjected to MARS-seq procedure to determine half-lives of polyadenylated RNAs; *n* = 2. (**C**) Changes in half-lives of multi- and single-exon mRNAs upon expression of HA-NSP1; *n* = 2. (**D**) HEK293 cells were transfected with plasmids encoding Renilla luciferase (Rluc) reporter genes either with or without intron in the 5′UTR (upper panel) along with plasmids encoding for eGFP or HA-NSP1. After 24 h, the relative expression of both reporters was calculated and plotted; *n* = 3, bars represent SE.

It was recently reported that endogenous mRNAs undergo destabilization in SARS-CoV-2-infected cells (9,10). In the case of SARS-CoV-1, this role has been attributed to NSP1, the first protein encoded by the viral genome (20,21). To test if NSP1 encoded by SARS-CoV-2 is sufficient to induce mRNA degradation, we transfected HEK293 cells with a plasmid encoding for HA-NSP1 and isolated polyadenylated mRNAs after 24 h of expression. Indeed, the expression of NSP1 significantly reduced the levels of polyadenylated RNAs in cells (Figure 1A). To directly examine the effect of NSP1 on mRNA stability, we measured the half-lives of cellular mRNAs via time-course actinomycin D treatment followed by RNA-seq (22). Indeed, we found that the expression of NSP1 strongly reduced the estimated average half-life of mRNAs from 12 to 3.5 h (Figure 1B). Upon detailed mRNA analysis, we noticed that the magnitude of the effect on mRNA degradation differed according to the presence of introns: while the stability of multi-exon mRNAs was highly sensitive to NSP1, intron-less transcripts were relatively resistant (Figure 1C). Genes binned according to their mRNA half-lives confirmed that NSP1 exerted a stronger effect on the multi-exon mRNAs, 54.5% of which exhibited movement over at least two bins as compared to 37.3% of single-exon mRNAs that exhibited similar behavior in NSP1-expressing cells (Figure S1A, B). We validated these half-life estimations by prolonged actinomycin D treatments measuring spliced endogenous mRNAs and histones, which are representatives of single-exon mRNAs, by an orthogonal RT-qPCR approach (Figure S1C, D respectively). We also employed Renilla (Rluc) reporter gene without or with an intron placed in its 5′UTR (Figure 1D, upper panel) that is spliced out during mRNA maturation (Figure S1E). The intron-encoding gene showed greater sensitivity to the expression of NSP1, both in protein and mRNA stability levels (Figure 1D and Figure S1F, G). Since viral mRNAs are intron-less, this observation provides a plausible explanation of how the virus ensures the relative stability of its own mRNAs. However, a major class of anti-viral genes, such as interferon-alpha and beta, are all intron-less. It was reported that SARS-CoV-2 represses these genes mainly at the translation level (8), suggesting that the translation machinery of infected cells must distinguish, among intron-less transcripts, between viral and host mRNAs. Overall, these results suggest that the expression of NSP1 leads to the global destabilization of preferentially the multi-exon host mRNAs and indicate that it may involve additional levels of repression.

**Figure 2.**
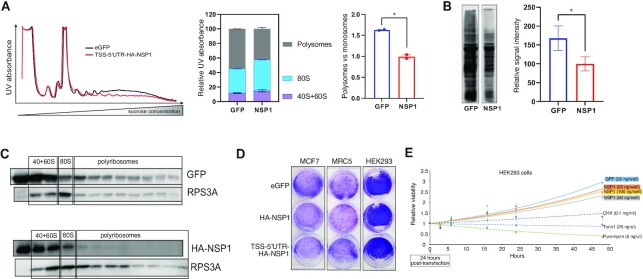
The impact of NSP1 on translation and survival. (**A**) HEK293 cells were transfected with plasmids encoding HA-NSP1 or eGFP, as a control. After 24 h, the cells were collected, lysed and subjected to polysomal profiling. The left panel displays continuous UV absorbance of both gradients of one out of two independent experiments. The middle panel shows the signal distribution between the different grouped fractions. The right panel directly compares the polysomal vs. monosomal fractions. In all panels, bars are SD of *n* = 2. See also Figure S2A for analysis of MCF7 cells. (**B**) HEK293 cells were transfected with plasmids encoding either eGFP or HA-NSP1. After 24 h, puromycin (10 μg/ml) was added for 5 min, and the cells were collected on ice, lysed and 50 μg of total lysates were resolved on 10% SDS-PAGE and probed with anti-puromycin antibodies. The relative signals obtained from the different lanes were analyzed and plotted on the right panel; *n* = 4, bars represent SD. See also Figure S2B for the original images and S2C for expression of specific proteins. (**C**) MCF7 cells were transfected with plasmids encoding either eGFP or HA-NSP1 and after 24 h lysed and subjected to polysomal isolation. The total proteins extracted from the collected polysomal fractions were separated on SDS-PAGE and probed to detect the indicated proteins. (**D**) MCF7, MRC5 and HEK293 cells were grown in 12-well dishes, transfected with either eGFP or HA-NSP1 (200 ng/well) and allowed to grow for additional 2 days, after which they were fixed and stained. These images are representative parts of Figure S2D. (**E**) HEK293 cells were seeded in 96-well plates and transfected with the indicated amounts of plasmids encoding for either eGFP or HA-NSP1. Twenty-four hours post-transfection (*t* = 0), the cells were subjected to proliferation assay at the indicated time points. As controls, cells were treated with known translation inhibitors at the indicated concentrations; *n* = 3, bars represent SE. See also Figure S2E for a similar analysis of MCF7 and Vero cells.

NSP1 was reported to interact with the host ribosome near the entry channel and inhibit translation due to physical hindrance to the ribosome-mRNA interaction (7,8,23). To test the effect of NSP1 on cellular translation, we performed polysome profiling of cells transfected with either HA-NSP1 or eGFP. Indeed, polysomal profiles of NSP1-expressing cells exhibited diminished polysomal fractions with an apparent enhancement of the 80S fractions (Figure 2A and Figure S2A), indicating reduced translation. The effect of NSP1 on translation was further examined by puromycin incorporation assay. Puromycin is a structural analog of aminoacylated-tRNA (aa-tRNA), which leads to premature termination of translation and labeling of nascent polypeptides. HEK293 cells were transfected with HA-NSP1 or GFP and subjected to a pulse of puromycin (10 μg/ml) for 5 min and subsequent western blotting using anti-puromycin antibodies. The results revealed a moderate loss of nascent polypeptide labeling upon NSP1 expression (Figure 2B and Figure S2B, C), confirming attenuated translation. Interestingly, we found that NSP1 protein was mainly present in the non-translating and initiating fractions of the polysomal gradient and was devoid from the fractions of actively translated mRNAs (Figure 2C). Taken together, these observations suggest that NSP1 may reduce the efficiency of the translation initiation step.

As translation is linked to cell proliferation and survival, we examined cell growth after transfection of either NSP1 or GFP, anticipating that the reduced translation will negatively affect the proliferation ability of the cells. However, we detected only minor growth defects in the NSP1-expressing cells (Figure 2D and Figure S2D). To confirm these findings, we monitored the cellular viability for up to 48 h post-transfection with HA-NSP1 and compared it to the effect of known translational inhibitors, such as Torin1, puromycin, or cycloheximide. We found that the expression of NSP1 did not significantly impact the growth kinetics, while the application of even low amounts of known translation inhibitors has markedly reduced it (Figure 2E and Figure S2E). Cumulatively, these findings uncover the relative impact of NSP1 on cellular translation and mRNA stability, and suggest that, at least within the tested time window, it does not significantly interfere with cellular proliferation.

### A precisely positioned RNA sequence motif confers resistance of viral mRNAs to NSP1-mediated inhibition of translation

**Figure 3.**
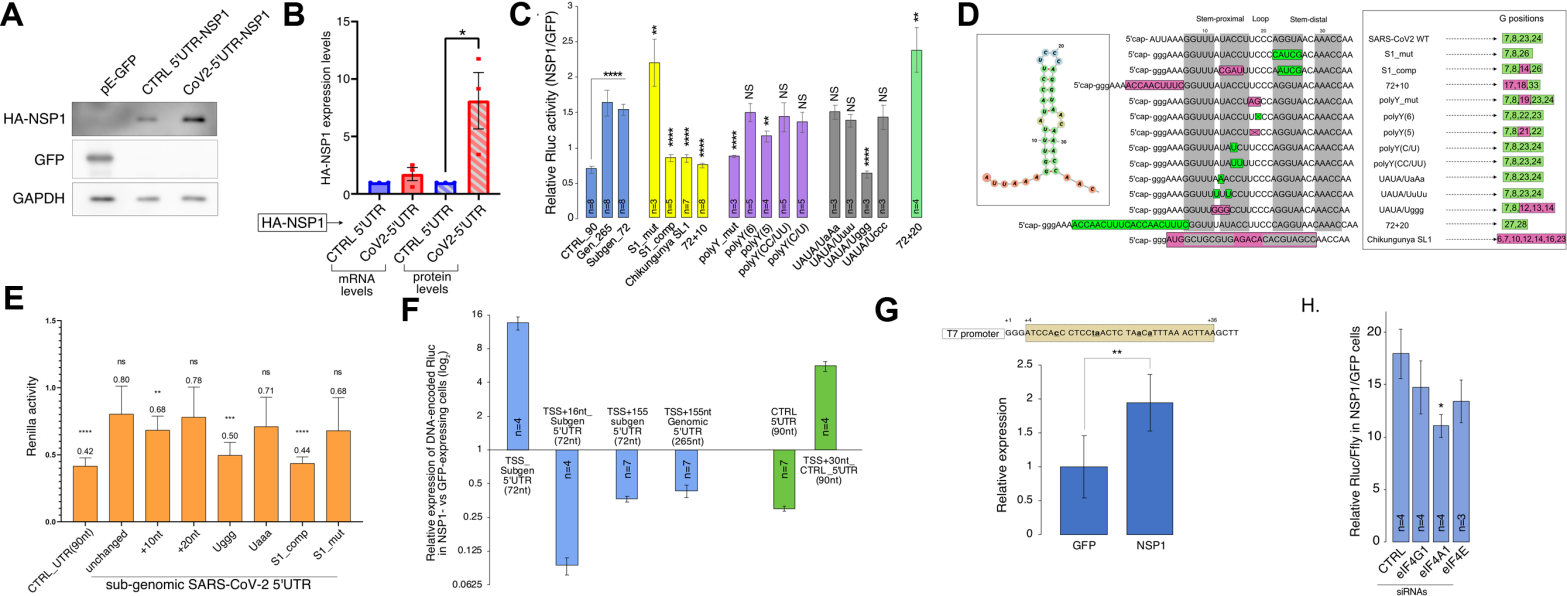
Identification of RNA sequence that protects from NSP1-mediated repression. (**A**) MRC5 cells were transfected with plasmids encoding for eGFP, control 5′UTR-HA-NSP1 or CoV2-5′UTR-HA-NSP1 with its native 5′UTR immediately following the TSS (which is termed HA-NSP1 in this study). After 24 h, the cells were collected and 50 μg from the extracted total proteins were resolved on 9% SDS-PAGE and probed to detect the indicated proteins. (**B**) MRC5 cells were transfected with plasmids encoding the control 5′UTR-HA-NSP1 or CoV2-5′UTR-HA-NSP1, as detailed in (A), harvested after 24 h and split into two equal parts. One part was subjected to RNA isolation and subsequent RT-qPCR analysis to determine mRNA levels. The second part of each harvest was dedicated to the resolution of total proteins (50 μg) on SDS-PAGE and detection of the indicated proteins. Relative values of each biological repeat (*n* = 3) were plotted, the bars represent SE. (**C**) Impact of NSP1 on the expression of reporter mRNAs. MRC5 cells were transfected with plasmids encoding either eGFP or TSS-5′UTR-HA-NSP1. After 20 h, the indicated mRNA reporters bearing both 5′cap and poly(A) tail were transfected, and the luciferase activity was measured 7 h later. The number of independent biological repeats is indicated for each reporter, the bars indicate SE. *P*-values indicated for the blue columns refer to the reporter bearing the control 5′UTR (CTRL_90), while for the rest of the columns it refers to the reporter encoding the sub-genomic SARS-CoV2 5′UTR (Subgen_72). (**D**) Detailed representation of the site-directed mutagenesis. The leftmost panel represents the SL1 element's structure, as folded by ViennaRNA software. The middle panel shows detailed schemes representing the manipulations done on the 5′cap-proximal region of the viral 5′UTR. Changed moieties are squared; green squares imply no change to expression, pink squares imply reduced expression, grey shades mark the nucleotides participating in the stem structures. The rightmost panel shows the positions of guanosine moieties within the corresponding sequences relative to the 5′cap. The green color indicates unperturbed expression, while new locations of the ‘G’ moieties that reduce expression are indicated in pink. (**E**) *In-vitro* transcribed Rluc mRNA reporters described in (C) bearing both 5′caps and poly(A) tails were added to rabbit reticulocyte lysates (RRL) pre-incubated with either purified HA-NSP1 or BSA. Rluc activity was tested after 60 min of incubation. The graph shows the relative ability of NSP1 to inhibit the different mRNA reporters; *n* = 3, bars represent SD. (**F**) MRC5 cells were co-transfected with i) plasmid encoding the indicated configurations of 5′UTRs fused to the reporter Rluc gene and ii) plasmid encoding for either HA-NSP1 or eGFP. The cells were harvested after 48 h and subjected to the examination of Rluc activity; the number of biological repeats (*n*) is indicated separately for each combination; bars represent SE. (**G**) DNA template for *in vitro* transcription encoding the control plasmid-derived 5′UTR was subjected to site-directed mutagenesis in order to create a guanosine-free region, as depicted on the upper panel. mRNAs transcribed from these templates were capped and transfected into MRC5 cells expressing either HA-NSP1 or eGFP. Luciferase activity was measured 7 h later and plotted in a relative manner; *n* = 6, bars represent SD. (**H**) MRC5 cells were transfected with the indicated siRNAs and after 48 h transfected again with plasmids encoding (i) either HA-NSP1 or eGFP, (ii) TSS-subgen_5′UTR-Rluc and, (iii) Firefly (Ffly). After additional 24 h, luminescence was tested and Rluc values were normalized by Ffly signal and compared to cells transfected with the control non-targeting siRNAs for statistical analysis; *n* = 3 or 4, bars represent SE.

Viral mRNAs produced in SARS-CoV-2-infected cells bear either long genomic (265-nt long) or shorter sub-genomic (∼70–75-nt long) 5′UTRs, the latter being produced via discontinuous transcription (6). The genomic 5′UTR of SARS-CoV-2 drives the translation of two first open reading frames (ORF1ab) that cumulatively yield 16 non-structural proteins. The NSP1-encoding construct used in this study until now encodes for a full genomic 5′UTR placed immediately after the transcription start site (TSS). Based on the previous reports (13), we anticipated that replacing the native viral 5′UTR with a control 5′UTR will expose NSP1 to self-repression. Indeed, this manipulation significantly reduced the protein levels of NSP1 (Figure 3A), with a relatively minor effect on the mRNA levels (Figure 3B), indicating that the genomic 5′UTR of SARS-CoV-2 grants resistance to auto-inhibition, most likely at the level of translation.

To understand which particular features within the genomic 5′UTR enable the observed resistance, we transfected capped mRNAs encoding the Renilla luciferase (Rluc) under the control of the SARS-CoV-2 genomic, sub-genomic or a control (90 nt-long) 5′UTRs into cells expressing either HA-NSP1 or GFP. While mRNA bearing the control 5′UTR was inhibited by NSP1, both the genomic and sub-genomic 5′UTRs not only conferred full protection but in fact boosted the reporter expression (Figure 3C, blue columns). To find the element enabling viral 5′UTRs to escape NSP1 inhibition, we focused on the first stem–loop structural element (SL1, Figure 3D, left panel), which was previously suggested to play a role in the propagation of SARS-CoV (12,13,24). First, we compromised its structure in the context of the sub-genomic (72-nt long) 5′UTR by introducing five point mutations in the distal part of the stem (S1_mut, Figure 3D). Surprisingly, disruption of this element did not eliminate the protection from NSP1-mediated repression and even slightly enhanced it (S1_mut, Figure 3C, yellow columns), thus uncoupling the structural integrity of this element from NSP1-mediated repression. Notably, further alteration of the sequence comprising SL1 to restore the stem–loop structure significantly disrupted the protective effect against NSP1 (S1_comp mRNA, Figure 3C and D). These findings strongly indicate that the ability to oppose NSP1 inhibition does not rely on the secondary structure of the SL1 element but rather on its primary RNA sequence. To further examine if cap-proximal stem–loop structures may protect mRNAs from NSP1, we exchanged the CoV-2-derived SL1 by an even longer stem–loop element derived from the Chikungunya virus (Chikungunya SL1, Figure 3C, D). This structural element was very inefficient in protecting the reporter transcript, indicating that cap-proximal secondary structures are unlikely to relieve NSP1-mediated inhibition. Importantly, insertion of 10 nt between the cap and the SL1 element also reduced the mRNA expression in the NSP1 presence (72 + 10 mRNA, Figure 3C, D), potentially indicating the importance of the location of this element relative to the 5′end. Taken together, these results suggest that the sequence comprising the SL1 element and its location relative to the 5′cap are critical features for resisting NSP1 inhibition, while the secondary structure of this element appears to be dispensable.

To understand the features necessary for mRNA protection in detail, we focused on two elements in the sequence comprising the SL1 element: a stretch of seven consecutive pyrimidines located in the proximal part of the stem and loop (nt. 15–21, Figure 3D), and a UAUA motif immediately preceding this polypyrimidine stretch (nt. 11–14). First, we examined the polypyrimidine stretch because its disruption (in the SL1_comp construct) greatly reduced the protection from NSP1. By manipulating this element (Figure 2C, purple columns), we found that: i) introduction of two purine residues (AG) in the middle of the stretch significantly reduced mRNA protection (see polyY_mut); ii) shortening the stretch from 7 to 5 pyrimidines mildly impaired the protection (see polyY(5)); and, iii) the precise pyrimidine sequence at the beginning of the stretch had no effect (see polyY(CC/UU) and polyY(C/U)). These results indicated that the length of the polypyrimidine stretch is important for NSP1 protection.

We next mutated the UAUA sequence to yield UAaA, UuUu, Uggg or Uccc. We found that only the insertion of guanosines turned the mRNA extremely sensitive to NSP1-mediated repression (see UAUA/Uggg, Figure 3C, D). In this mutant, the polypyrimidine stretch was intact, suggesting that it is insufficient to grant NSP1 resistance. These results suggest that the presence of guanosines, but not adenosines uridines or cytosines, in the SL1 region may predispose mRNAs to NSP1-mediated repression. To test this further, we mapped the precise locations of guanosines in the different mRNA variants that we tested above (Figure 3D, right panel). While the original viral 5′UTR lacks guanosines between positions 9–22 (inclusive), all mutant 5′UTR versions that were repressed by NSP1 (e.g. S1_comp, WT + 10, etc.) involved the insertion of guanosines between these precise positions (magenta squares). Intriguingly, manipulations that did not alter the relative positions of the guanosine residues (green squares) rendered the respective mRNAs resilient to NSP1. To test if the precise positioning of guanosines could play a role in NSP1 resistance, we duplicated the 10 nt of the 72 + 10 mRNA to create a new, 72 + 20, version. Although no new sequences were introduced into this 5′UTR version, the repetitive insertion of the 10-nt sequence pushed the existing guanosines from positions 17, 18 to 27, 28 relative to the 5′-cap (Figure 3D). Strikingly, this manipulation fully restored the NSP1 resistance (Figure 3C, 72 + 20). Since these 10 nucleotides, which are guanosine-free, were already present in the 72 + 10 mRNA, this result indicates that this sequence *per se* is insufficient to resist NSP1 but important to create a cap-proximal guanosine-free stretch. To test if 5′cap-proximal guanosine-free stretches are conserved in coronaviruses, we mapped G-free regions in the 5′UTRs of several alpha- and beta-coronaviruses (Figure S3A). Indeed, we found that multiple CoV-derived 5′UTRs encode for cap-proximal guanosine-free stretches, indicating the conservation and, therefore, importance of this feature. Notably, we recapitulated this phenomenon using a cell-free rabbit reticulocyte lysate (RRL) *in vitro* translation system (Figure 3E). We found that this effect does not stem from differences in the mRNA stability in either RRL or transfected cells (Figure S3B, C), indicating that this regulation occurs most likely at the level of translation, as guanosine residues located between the positions 11–20 relative to the 5′cap structure of endogenous mRNAs do not alter mRNA stabilities in the presence of NSP1 (Figure S3D). These results suggest cap-proximal guanosine-free sequences are crucial to grant resistance to NSP1-mediated repression.

To test these conclusions further, we decided to apply them on plasmid-encoded genes, which are efficiently repressed by NSP1 (e.g. Figure 1D). We constructed a DNA-encoded reporter gene preceded by the sub-genomic version of the viral 5′UTR immediately downstream the TSS in order to preserve the guanosine-free state at the beginning of the transcript. Indeed, we found that the precise location of the viral 5′UTR sequence not only fully protected the respective mRNA from NSP1 repression but, in fact, enhanced its expression by ∼13-fold in NSP1-expressing cells (Figure 3F, TSS_subgen 5′UTR). Moving the viral 5′UTRs away from the TSS by either 16 or 155 bases strongly reduced the protein activity of the respective genes and promoted vulnerability to NSP1 inhibition, both in the context of genomic and sub-genomic 5′UTRs (Figure 3F, blue columns). These results strongly support the notion that the precise location of the viral 5′UTR relative to the cap structure is crucial to escape NSP1-mediated repression. To examine whether the first 30-nt of the viral 5′UTR encoding the guanosine-free stretch of nucleotides are sufficient for this effect, we inserted them after the TSS of the control 5′UTR, which is otherwise NSP1-sensitive. This manipulation not only abolished its inhibition by NSP1 but also boosted its expression by ∼5-fold (Figure 3F, green columns). Furthermore, the substitution of guanosines between positions 4 and 36 in the non-viral control 5′UTR resulted in a complete escape from the NSP1-mediated repression (Figure 3G), similar to the 5′UTRs encoded by CoV-2 (see Figure 3C, blue columns). Altogether, these findings support the notion that guanosine-free sequences precisely located relative to the 5′end are sufficient to confer NSP1 resistance.

Lastly, we assumed that successful translation in NSP1-expressing cells might require the unwinding of the secondary structure of SL1 in order to expose its primary RNA sequence. We, therefore, knocked-down the different subunits of the eIF4F complex and tested the impact of NSP1 on the DNA construct encoding the sub-genomic 5′UTR. Knockdown efficiencies were validated at the mRNA and protein levels (Figure S3E–G). Interestingly, although knocking-down certain components of this complex impacted other, non-targeted subunits (Figure S3F), we found that downregulation of the eIF4A1 RNA helicase component had the strongest negative effect on the ability of NSP1 to promote the expression of this gene (Figure 3H), supporting the notion of the necessity to unwind the viral SL1 structure to enable efficient protection from NSP1 inhibition.

### The genomic viral 5′UTR enables 5′cap-independent translation, which is refractory to NSP1 inhibition.

**Figure 4.**
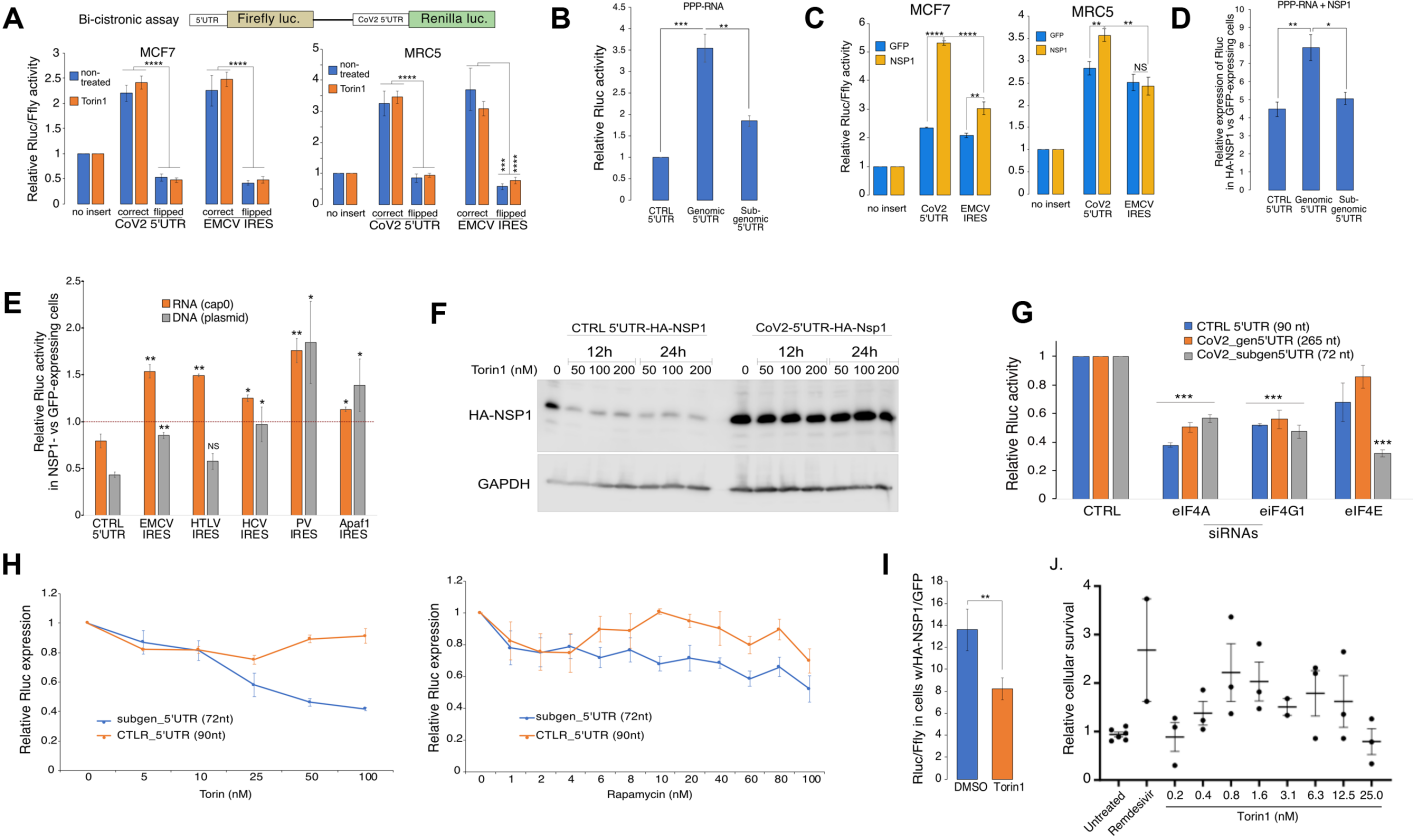
The translational features of SARS-CoV-2 5′UTRs. (**A**) Upper panel: schematic representation of the bi-cistronic assay. MCF7 (left panel) or MRC5 (right panel) cells were transfected with bi-cistronic plasmids encoding for the SARS-CoV-2 genomic 5′UTR or EMCV-derived IRES element as a positive control. Both elements were introduced in their correct or flipped (i.e. anti-sense) configuration as a control for non-specific activity. Cells were grown for 24 h in standard medium (NT) or supplied with Torin1 (25 nM), after which Rluc and Ffly activities were assayed; *n* = 4, bars represent SE. (**B**) MRC5 cells were transfected with the indicated uncapped (PPP) *in-vitro* transcribed mRNAs bearing poly(A) tails. Rluc activity was tested 7 h later and is presented relative to the control plasmid-derived 5′UTR; *n* = 4, the bars represent SE. (**C**) MCF7 and MRC5 cells were transfected with i) the indicated bi-cistronic plasmids and ii) plasmid encoding either HA-NSP1 or eGFP. Luminescence was assayed 24 h later, *n* = 4, the bars represent SD. (**D**) MRC5 cells were transfected with plasmids encoding either HA-NSP1 or eGFP. After 24 h, uncapped RNAs with poly(A) tails encoding for indicated 5′UTRs and a downstream Rluc gene were transfected and Rluc activity was assayed 7 h later; *n* = 4, bars represent SE. (**E**) Grey columns: MRC5 cells were co-transfected with plasmids encoding i) either HA-NSP1 or eGFP and ii) indicated IRES sequences cloned before the Rluc reporter gene. Orange columns: MRC5 cells were transfected with plasmids encoding either HA-NSP1 or eGFP and, after 24 h, with capped mRNAs *in vitro* transcribed from the indicated IRES-containing plasmids; *n* = 2, bars represent SD. (**F**) HEK293 cells were transfected with plasmids encoding HA-NSP1 preceded by either control or viral 5′UTR and grown for 40 h post-transfection, out of which Torin1 was added during the last 12 or 24 h at the indicated concentrations. After harvest, total protein lysates were separated on 9% SDS-PAGE and probed with anti-HA and anti-GAPDH antibodies. (**G**) MRC5 cells were transfected with the indicated siRNAs, and re-transfected after 65 h with *in-vitro* transcribed mRNA reporters bearing both 5′caps and poly(A) tails. Rluc activity was tested 7 h after the second transfection and presented relatively to the cells expressing the control siRNA; *n* = 4, bars represent SE. (**H**) MRC5 cells were transfected with the indicated *in-vitro* transcribed mRNA reporters bearing both 5′caps and poly(A) tails. After 2 h, Torin1 (left panel) or Rapamycin (right panel) were added to yield the indicated concentrations and Rluc activity was tested after additional 5 h; *n* = 3, the bars represent SE. (**I**) MRC5 cells were transfected with the mix of plasmids encoding (i) either HA-NSP1 or eGFP, (ii) TSS-subgen_5′UTR-Rluc and, (iii) Firefly (Ffly). After 4 h of transfection, the medium was replaced and either DMSO or Torin1 (25 nM) were added. The cells were collected after 24 h, lysed and subjected to luminescence analysis. Rluc values were normalized against Ffly signal, *n* = 3, the bars represent SE. (**J**) Vero E6 cells were treated with the indicated final concentrations of Torin1 for 1 h, infected with SARS-CoV-2 at MOI = 0.01–0.015 and incubated for 3 days in the presence of Torin1. Following this time, the relative cytopathic effect of the virus was tested. As a positive control, cells were treated with Remdesivir (0.3 mM), for negative relative control cells were not treated prior to infection; *n* = 2 or 3, bars indicate SE.

Next, we investigated the translational properties of the genomic (265-nt) and sub-genomic (72-nt) 5′UTRs of SARS-CoV-2. We reasoned that since the cap-proximal sequence that grants protection from NSP1-mediated repression is present in both versions, the longer 5′UTR may encode additional translational features. Particularly, we tested its capacity to promote cap-independent translation initiation, a feature common to multiple positive single-stranded RNA viruses. To this end, we subcloned the genomic 5′UTR into a bi-cistronic system (Figure 4A, upper panel) and tested its cap-independent translational activity in MCF7 cells. Interestingly, the presence of the genomic 5′UTR markedly enhanced the expression of the downstream Rluc gene, in a manner comparable to the well-characterized EMCV-derived IRES element (Figure 4A, left, blue columns). We observed a similar effect in MRC5 cells (Figure 4A, right panel, blue columns), suggesting that this feature is not cell-type specific. Notably, neither element showed enhanced activity when cloned in the opposite (flipped) configuration, indicating the importance of the primary RNA sequences. Translation of both transcripts was resistant to Torin1, a pharmacological agent that limits the availability of eIF4E via 4E-BP phosphorylation (Figure S4D) and inhibits cap-dependent translation (Figure 4A, orange columns in both graphs), indicating their potential ability to sustain protein synthesis even upon inhibition of cap-dependent initiation. We confirmed that the presence of the viral 5′UTR did not lead to higher mRNA expression of the second Rluc ORF (Figure S4A), ruling out the possibility that it has a cryptic promoter activity. Next, we transfected *in vitro* transcribed uncapped reporter mRNAs bearing different viral 5′UTRs into cells and assayed the protein expression encoded by the corresponding transcripts. Indeed, we observed a relatively enhanced (∼3.5-fold) expression of the reporter mRNA bearing the genomic 5′UTR version (Figure 4B), which disappeared when the sequence was cloned in an anti-sense configuration (Figure S4B), indicating sequence specificity.

To investigate the effect of NSP1 on cap-independent translation, we initially used the bi-cistronic tests and found that NSP1 enhanced the cap-independent translation directed by the viral genomic 5′UTR (Figure 4C). However, the bi-cistronic assays are not an ideal tool to study the effect of NSP1 on cap-independent translation. First, the long bi-cistronic transcript is transcribed by RNA polymerase II and is, therefore, capped. Second, being synthesized in the nucleus, it is exposed to possible additional effects of NSP1, such as interference with the nuclear export (25). To directly test the impact of NSP1 on cap-independent expression, we transfected uncapped mRNAs into cells expressing NSP1 and found that it significantly boosted protein production from all tested transcripts, even those not encoding for viral 5′UTRs (Figure 4D). Notably, these uncapped mRNAs were not more stable in the context of NSP1 (Figure S4C). These results indicate that NSP1 may non-specifically enhance cap-independent expression. To test this assumption further, we employed a reporter gene fused to different IRES elements that were transfected into NSP1-expressing cells either as capped mRNAs or as plasmids. We found that when introduced as capped mRNAs, all tested IRES elements moderately enhanced the expression of their corresponding transcripts in the presence of NSP1 (Figure 4E, orange columns), while not displaying preferential mRNA stability in NSP1-expressing cells (Figure S4C). However, when the corresponding mRNAs were transcribed in cells, the impact of NSP1 varied (grey columns) and certain IRES elements were inhibited, implying possible differential effects of NSP1 on IRES-encoding transcripts, as was reported previously (13,26).

Our findings suggest that the expression of NSP1 itself, which is preceded by the genomic 5′UTR, has two important protective features. First, the cap-proximal guanosine-deficient element protects it from self-repression. Second, the cap-independent activity of the genomic 5′UTR may enable expression upon inhibition of cap-dependent translation. To examine the extent by which these features affect NSP1 expression, as a proxy of viral genes encoded by ORF1ab, we employed two versions of NSP1: one bearing the native genomic 5′UTR correctly positioned relative to the 5′cap and the second under a control 5′UTR. Upon transfection into cells and application of Torin1, we found that in contrast to the control 5′UTR, the native 5′UTR not only enhanced NSP1 expression but also retained its high levels despite prolonged treatments with Torin1 (Figure 4F).

### **The sub-genomic 5**′**UTR of SARS-CoV-2 exhibits a high dependency on eIF4E**.

Next, we tested the sensitivity of the viral 5′UTRs to the knock-down of eIF4F components (Figure S3E, F). Knock-down of either eIF4A1 or eIF4G1 reduced the translatability of all reporters, whereas knock-down of the cap-binding eIF4E subunit had a differential effect on the viral 5′UTRs (Figure 4G). While the genomic 5′UTR exhibited relative insensitivity to eIF4E deficiency, consistent with its ability to resist attenuation of cap-dependent translation, the expression directed by the sub-genomic 5′UTR was significantly reduced. To examine further the sensitivity of the sub-genomic version of the viral 5′UTR to inhibition of cap-dependent translation, we applied two mTOR inhibitors, Torin1 and Rapamycin that affect 4E-BP phosphorylation (Figure S4D, E), and elucidated their effects on the translation of the reporter mRNA driven by the sub-genomic 5′UTR. Indeed, transcripts bearing this 5′UTR were remarkably vulnerable to both inhibitors (Figure 4H), suggesting that translation mediated by this sub-genomic 5′UTR heavily relies on cap-dependent initiation. In light of this dependency, we examined the impact of NSP1 on the expression directed by this 5′UTR upon Torin1 treatment. Interestingly, while NSP1 efficiently boosted the expression of DNA-encoded genes bearing viral sub-genomic 5′UTR, this ability was reduced upon Torin1 treatment (Figure 4I), confirming the important role of cap-dependent translation in the expression of transcripts encoding for sub-genomic 5′UTRs, even in the context of NSP1.

Considering the overall negative impact of inhibition of cap-dependent translation on the expression of sub-genomic 5′UTRs, we tested the effect of Torin1 treatment on virus propagation in cells. For this purpose, we treated Vero E6 cells with different concentrations of Torin1, infected them with SARS-CoV-2 and monitored the cytotoxic effect of the virus after 72 h. We found that mTOR inhibition enhanced the survival of the infected cells at relatively low doses (0.8–12.5 nM), while at higher concentrations it was toxic for cells (Figure 4J). These findings suggest that moderate inhibition of cap-dependent translation may interfere with viral propagation in cells.

## DISCUSSION

NSP1 protein encoded by alpha- and beta-coronaviruses plays a central role in the virus infectivity in general and the ability to control the host translation machinery in particular (27). By binding the mRNA entry channel of the small ribosomal subunit, NSP1 of SARS-CoV-1/2 both hinders the translation of cellular mRNAs and induces their degradation by a still unclear mechanism. In this study, we elucidated the molecular features that enable SARS-CoV-2 to hijack host translation to prevent efficient expression of host genes and, at the same time, to enable its own mRNAs to avoid these inhibitory mechanisms. We found that the mere expression of NSP1 in cells, out of the viral context, is sufficient to target the vast majority of the host mRNAs for degradation, consistent with recent studies (9–11). We further reveal that while NSP1 induces robust degradation of spliced mRNAs, single-exon mRNAs remain relatively less sensitive. Interestingly, interferon-alpha and beta genes that comprise the first line of the host anti-viral response, are intron-less and, therefore, likely to be resilient to NSP1-induced mRNA degradation. A potential explanation of this phenomenon may be linked to the presence of the exon junction complex (EJC) in spliced mRNAs, which is known to facilitate the recruitment of ribosomes (28), where NSP1 resides. This dependency of mRNA degradation on recruitment to ribosomes is supported by the relatively reduced vulnerability of long non-coding RNAs to NSP1-induced degradation (Figure S1H). Although this hypothesis has to be further examined, the presented findings expand the understanding of the differential impact of NSP1 encoded by beta-coronaviruses on the decay of host mRNAs, as was reported for MERS-CoV (29) and SARS-CoV-1 (30,31).

To further limit the expression of the host genes, SARS-CoV-2 has evolved an effective suppression strategy at the translation level, which hampers the entrance of mRNAs into the ribosome (7,8,23). We demonstrate that the expression of NSP1 does not entirely shut down the host translation, as evident from the polysomal profiles and puromycin labeling experiments (Figure 2A, B), but rather attenuates the translation initiation step, consistent with previous reports (23,26). In support of that, we found that NSP1 is enriched in the initiating polysomal fractions (Figure 2C), leading to enhanced RNA content in the fractions representing 80S (Figure 2A and Figure S2A). These findings agree with recent reports of relatively unperturbed translation elongation dynamics in both infected cells (10) and upon NSP1 expression (11). Surprisingly, despite the prominent effects of NSP1 on host gene expression, we found only mild cytotoxic effects of NSP1 expression in multiple tested cell types (Figure 2D, E and Figure S2D, E). While the precise roots of this phenomenon remain to be understood, it is likely to be related to its relatively moderate effect on translation, which is important to ensure successful viral propagation. Noteworthy, these effects of NSP1 on host gene expression and survival might be modulated in the context of viral infection.

Overall, we show that SARS-CoV-2 employs at least three strategies to promote efficient translation of its own mRNAs. First, by inducing degradation of most cellular mRNAs (Figure 1B, C), NSP1 reduces the competition of the viral intron-less transcripts with cellular mRNAs for accessing the ribosomes. Second, the guanosine-free region within the SARS-CoV-2 5′UTRs is critical to confer protection from NSP1-mediated inhibition of translation. A detailed characterization of this element revealed that it overlaps the SL1 element, as reported (13,32–34), but our results suggest that the primary sequence mediates the protective effect rather than the secondary structure it creates. This finding is in agreement with a recently published study that reported the importance of the primary sequence for NSP1 resistance (35). Furthermore, our results are supported by the strong dependency of viral transcripts on eIF4A (Figure 3H and (36,37)) that might be needed to unwind the SL1 structure and expose its sequence. We show that both depletion of guanosines and the proximity to the 5′cap are the most critical features of this element (Figure 3C, D), which are conserved among several coronaviruses (Figure S3A). Fulfilling these requirements rendered heterologous genes fully protected from NSP1-mediated repression (Figure 3F, G). While the mechanism underlying the protective ability of this element is still an open question, it is tempting to speculate that its precise location relative to the 5′-cap structure is linked to the cap-binding by eIF4F. Considering a previous report of SARS-CoV-1 NSP1 binding to SL1 (12), another possibility is a preferential binding of NSP1 itself to the G-less motif. However, we failed to detect any RNA binding activity of NSP1 both *in vitro* and in transfected cells (data not shown), as recently reported (13). Third, we identified cap-independent translation activity of the genomic version of the viral 5′UTR, which bypassed reduced eIF4E availability in a manner comparable to known IRES elements (Figure 4A). This feature may enable cap-independent expression of the first two viral ORFs encoding 16 non-structural viral proteins as demonstrated for NSP1 (Figure 4F). Importantly, the sub-genomic versions of the viral 5′UTR are highly eIF4E-dependent (Figure 4G-H), which also reflects on the ability of NSP1 to support the expression of mRNAs encoding these 5′UTRs (Figure 4I). This phenomenon may contribute to the sensitivity of SARS-CoV-2 to inhibitors of eIF4E, at least in culture (Figure 4J). Notably, the efficacy of mTOR targeting as a potential anti-COVID19 treatment is currently under debate (38–41), and our findings support the possible efficacy of such therapy. However, additional experiments with carefully quantified viral titers are required to validate the potential anti-viral activity.

The ability of the genomic 5′UTR of SARS-CoV-2 to mediate cap-independent translation initiation is rather intriguing, mainly because all viral transcripts bear 5′cap structures. This feature might be necessary at the initial stages of infection, which provoke interferon response, leading to the inhibition of cap-dependent translation (1,42). Under these conditions, the genomic 5′UTR may be of particular importance, promoting cap-independent translation of ORF1ab and synthesis of multiple non-structural proteins necessary to counteract the host defense (32) and support the exploitation of the cellular machineries. Interestingly, we found that a mere expression of NSP1 promotes cap-independent translation, enabling particularly high translational yields from uncapped mRNAs (Figure 4D). These findings corroborate the notion that SARS-CoV-2 may be well prepared for hijacking the host translational machinery under conditions of inhibited cap-dependent translation. However, at the later stages of infection, when discontinuous transcription leads to the synthesis of multiple viral mRNAs bearing short sub-genomic 5′UTRs that strongly depend on eIF4E availability, cap-dependent initiation might become necessary for viral proliferation. This might be an Achilles heel of the viral replication and the reason for the observed sensitivity of SARS-CoV-2 to pharmacological mTOR inhibitors (41). In this context, it would be interesting to test in future studies the ability of SARS-CoV-2 to manipulate the availability of the eIF4F complex along the infection cycle. Taken together, this study highlights the roles of NSP1 and the viral 5′UTRs in the translation regulation exerted by the virus. These features could be further exploited as major vulnerabilities of SARS-CoV-2 and are, therefore, excellent targets for general therapeutic applications.

## DATA AVAILABILITY

All data regarding the genome-wide determination of mRNA stability presented in Figure 1 and Figure S1 are publicly available in the GEO database (GSE193732).

## Supplementary Material

gkac615_Supplemental_Files

## References

[1] Schoggins J.W. , Wilson S.J., Panis M., Murphy M.Y., Jones C.T., Bieniasz P., Rice C.M. A diverse range of gene products are effectors of the type I interferon antiviral response. Nature. 2011; 472:481–485. 21478870 10.1038/nature09907 PMC3409588

[2] Slobodin B. , Dikstein R. So close, no matter how far: multiple paths connecting transcription to mRNA translation in eukaryotes. EMBO Rep. 2020; 21:e50799. 32803873 10.15252/embr.202050799 PMC7507372

[3] Martin S. , Saha B., Riley J.L. The battle over mTOR: an emerging theatre in host-pathogen immunity. PLoS Pathog. 2012; 8:e1002894. 23028309 10.1371/journal.ppat.1002894 PMC3441621

[4] Yokoyama T. , Machida K., Iwasaki W., Shigeta T., Nishimoto M., Takahashi M., Sakamoto A., Yonemochi M., Harada Y., Shigematsu H. et al. HCV IRES captures an actively translating 80S ribosome. Mol. Cell. 2019; 74:1205–1214. 31080011 10.1016/j.molcel.2019.04.022

[5] Mariano G. , Farthing R.J., Lale-Farjat S.L.M., Bergeron J.R.C. Structural characterization of SARS-CoV-2: where we are, and where we need to be. Front. Mol. Biosci. 2020; 7:605236. 33392262 10.3389/fmolb.2020.605236 PMC7773825

[6] Kim D. , Lee J.Y., Yang J.S., Kim J.W., Kim V.N., Chang H. The architecture of SARS-CoV-2 transcriptome. Cell. 2020; 181:914–921. 32330414 10.1016/j.cell.2020.04.011 PMC7179501

[7] Schubert K. , Karousis E.D., Jomaa A., Scaiola A., Echeverria B., Gurzeler L.-A., Leibundgut M., Thiel V., Mühlemann O., Ban N. SARS-CoV-2 Nsp1 binds the ribosomal mRNA channel to inhibit translation. Nat. Struct. Mol. Biol. 2020; 27:959–966. 32908316 10.1038/s41594-020-0511-8

[8] Thoms M. , Buschauer R., Ameismeier M., Koepke L., Denk T., Hirschenberger M., Kratzat H., Hayn M., Mackens-Kiani T., Cheng J. et al. Structural basis for translational shutdown and immune evasion by the Nsp1 protein of SARS-CoV-2. Science. 2020; 369:1249–1255. 32680882 10.1126/science.abc8665 PMC7402621

[9] Burke J.M. , Clair L.A.S., Perera R., Parker R. SARS-CoV-2 infection triggers widespread host mRNA decay leading to an mRNA export block. RNA. 2021; 27:1318–1329. 34315815 10.1261/rna.078923.121 PMC8522697

[10] Finkel Y. , Gluck A., Nachshon A., Winkler R., Fisher T., Rozman B., Mizrahi O., Lubelsky Y., Zuckerman B., Slobodin B. et al. SARS-CoV-2 uses a multipronged strategy to impede host protein synthesis. Nature. 2021; 594:240–245. 33979833 10.1038/s41586-021-03610-3

[11] Rao S. , Hoskins I., Tonn T., Garcia P.D., Ozadam H., Sarinay Cenik E., Cenik C. Genes with 5′ terminal oligopyrimidine tracts preferentially escape global suppression of translation by the SARS-CoV-2 Nsp1 protein. RNA. 2021; 27:1025–1045. 34127534 10.1261/rna.078661.120 PMC8370740

[12] Tanaka T. , Kamitani W., DeDiego M.L., Enjuanes L., Matsuura Y. Severe acute respiratory syndrome coronavirus nsp1 facilitates efficient propagation in cells through a specific translational shutoff of host mRNA. J. Virol. 2012; 86:11128–11137. 22855488 10.1128/JVI.01700-12 PMC3457165

[13] Tidu A. , Janvier A., Schaeffer L., Sosnowski P., Kuhn L., Hammann P., Westhof E., Eriani G., Martin F. The viral protein NSP1 acts as a ribosome gatekeeper for shutting down host translation and fostering SARS-CoV-2 translation. RNA. 2021; 27:253–264. 10.1261/rna.078121.120 PMC7901841 33268501

[14] Frey S. , Görlich D. Purification of protein complexes of defined subunit stoichiometry using a set of orthogonal, tag-cleaving proteases. J. Chromatogr. A. 2014; 1337:106–115. 24636567 10.1016/j.chroma.2014.02.030

[15] Slobodin B. , Han R., Calderone V., Vrielink J.A.F.O., Loayza-Puch F., Elkon R., Agami R. Transcription impacts the efficiency of mRNA translation via Co-transcriptional N6-adenosine methylation. Cell. 2017; 169:326–337. 28388414 10.1016/j.cell.2017.03.031 PMC5388891

[16] Vallejos M. , Carvajal F., Pino K., Navarrete C., Ferres M., Huidobro-Toro J.P., Sargueil B., López-Lastra M. Functional and structural analysis of the internal ribosome entry site present in the mRNA of natural variants of the HIV-1. PLoS ONE. 2012; 7:e35031. 22496887 10.1371/journal.pone.0035031 PMC3319624

[17] Jaitin D.A. , Kenigsberg E., Keren-Shaul H., Elefant N., Paul F., Zaretsky I., Mildner A., Cohen N., Jung S., Tanay A. et al. Massively parallel single cell RNA-Seq for marker-free decomposition of tissues into cell types. Science. 2014; 343:776–779. 24531970 10.1126/science.1247651 PMC4412462

[18] Kohen R. , Barlev J., Hornung G., Stelzer G., Feldmesser E., Kogan K., Safran M., Leshkowitz D. UTAP: user-friendly transcriptome analysis pipeline. BMC Bioinformatics. 2019; 20:154. 30909881 10.1186/s12859-019-2728-2 PMC6434621

[19] Dobin A. , Davis C.A., Schlesinger F., Drenkow J., Zaleski C., Jha S., Batut P., Chaisson M., Gingeras T.R. STAR: ultrafast universal RNA-seq aligner. Bioinformatics. 2013; 29:15–21. 23104886 10.1093/bioinformatics/bts635 PMC3530905

[20] Kamitani W. , Narayanan K., Huang C., Lokugamage K., Ikegami T., Ito N., Kubo H., Makino S. Severe acute respiratory syndrome coronavirus nsp1 protein suppresses host gene expression by promoting host mRNA degradation. Proc. Natl. Acad. Sci. U.S.A. 2006; 103:12885–12890. 16912115 10.1073/pnas.0603144103 PMC1568942

[21] Narayanan K. , Huang C., Lokugamage K., Kamitani W., Ikegami T., Tseng C.-T.K., Makino S. Severe acute respiratory syndrome coronavirus nsp1 suppresses host gene expression, including that of type I interferon, in infected cells. J. Virol. 2008; 82:4471–4479. 18305050 10.1128/JVI.02472-07 PMC2293030

[22] Slobodin B. , Bahat A., Sehrawat U., Becker-Herman S., Zuckerman B., Weiss A.N., Han R., Elkon R., Agami R., Ulitsky I. et al. Transcription dynamics regulate poly(a) tails and expression of the RNA degradation machinery to balance mRNA levels. Mol. Cell. 2020; 78:434–444. 32294471 10.1016/j.molcel.2020.03.022

[23] Lapointe C.P. , Grosely R., Johnson A.G., Wang J., Fernández I.S., Puglisi J.D. Dynamic competition between SARS-CoV-2 NSP1 and mRNA on the human ribosome inhibits translation initiation. Proc. Natl. Acad. Sci. U.S.A. 2021; 118:e2017715118. 33479166 10.1073/pnas.2017715118 PMC8017934

[24] Vankadari N. , Jeyasankar N.N., Lopes W.J. Structure of the SARS-CoV-2 Nsp1/5′-untranslated region complex and implications for potential therapeutic targets, a vaccine, and virulence. J. Phys. Chem. Lett. 2020; 11:9659–9668. 33135884 10.1021/acs.jpclett.0c02818

[25] Zhang K. , Miorin L., Makio T., Dehghan I., Gao S., Xie Y., Zhong H., Esparza M., Kehrer T., Kumar A. et al. Nsp1 protein of SARS-CoV-2 disrupts the mRNA export machinery to inhibit host gene expression. Sci. Adv. 2021; 7:eabe7386. 33547084 10.1126/sciadv.abe7386 PMC7864571

[26] Lokugamage K.G. , Narayanan K., Huang C., Makino S. Severe acute respiratory syndrome coronavirus protein nsp1 is a novel eukaryotic translation inhibitor that represses multiple steps of translation initiation. J. Virol. 2012; 86:13598–13608. 23035226 10.1128/JVI.01958-12 PMC3503042

[27] Narayanan K. , Ramirez S.I., Lokugamage K.G., Makino S. Coronavirus nonstructural protein 1: common and distinct functions in the regulation of host and viral gene expression. Virus Res. 2015; 202:89–100. 25432065 10.1016/j.virusres.2014.11.019 PMC4444399

[28] Nott A. , Le Hir H., Moore M.J. Splicing enhances translation in mammalian cells: an additional function of the exon junction complex. Genes Dev. 2004; 18:210–222. 14752011 10.1101/gad.1163204 PMC324426

[29] Lokugamage K.G. , Narayanan K., Nakagawa K., Terasaki K., Ramirez S.I., Tseng C.-T.K., Makino S. Middle east respiratory syndrome coronavirus nsp1 inhibits host gene expression by selectively targeting mRNAs transcribed in the nucleus while sparing mRNAs of cytoplasmic origin. J. Virol. 2015; 89:10970–10981. 26311885 10.1128/JVI.01352-15 PMC4621111

[30] Kamitani W. , Huang C., Narayanan K., Lokugamage K.G., Makino S. A two-pronged strategy to suppress host protein synthesis by SARS coronavirus Nsp1 protein. Nat. Struct. Mol. Biol. 2009; 16:1134–1140. 19838190 10.1038/nsmb.1680 PMC2784181

[31] Huang C. , Lokugamage K.G., Rozovics J.M., Narayanan K., Semler B.L., Makino S. SARS coronavirus nsp1 protein induces template-dependent endonucleolytic cleavage of mRNAs: viral mRNAs are resistant to nsp1-induced RNA cleavage. PLoS Pathog. 2011; 7:e1002433. 22174690 10.1371/journal.ppat.1002433 PMC3234236

[32] Banerjee A.K. , Blanco M.R., Bruce E.A., Honson D.D., Chen L.M., Chow A., Bhat P., Ollikainen N., Quinodoz S.A., Loney C. et al. SARS-CoV-2 disrupts splicing, translation, and protein trafficking to suppress host defenses. Cell. 2020; 183:1325–1339. 33080218 10.1016/j.cell.2020.10.004 PMC7543886

[33] Vora S.M. , Fontana P., Mao T., Leger V., Zhang Y., Fu T.-M., Lieberman J., Gehrke L., Shi M., Wang L. et al. Targeting stem–loop 1 of the SARS-CoV-2 5′ UTR to suppress viral translation and Nsp1 evasion. Proc. Natl. Acad. Sci. U.S.A. 2022; 119:e2117198119. 35149555 10.1073/pnas.2117198119 PMC8892331

[34] Shi M. , Wang L., Fontana P., Vora S., Zhang Y., Fu T.-M., Lieberman J., Wu H. SARS-CoV-2 Nsp1 suppresses host but not viral translation through a bipartite mechanism. 2020; bioRxiv doi:20 September 2020, preprint: not peer reviewed 10.1101/2020.09.18.302901.

[35] Bujanic L. , Shevchuk O., Kügelgen N., Kalinina A., Ludwik K., Koppstein D., Zerna N., Sickmann A., Chekulaeva M. The key features of SARS-CoV-2 leader and NSP1 required for viral escape of NSP1-mediated repression. RNA. 2022; 28:766–779. 35232816 10.1261/rna.079086.121 PMC9014875

[36] Müller C. , Schulte F.W., Lange-Grünweller K., Obermann W., Madhugiri R., Pleschka S., Ziebuhr J., Hartmann R.K., Grünweller A. Broad-spectrum antiviral activity of the eIF4A inhibitor silvestrol against corona- and picornaviruses. Antiviral Res. 2018; 150:123–129. 29258862 10.1016/j.antiviral.2017.12.010 PMC7113723

[37] Müller C. , Obermann W., Karl N., Wendel H.-G., Taroncher-Oldenburg G., Pleschka S., Hartmann R.K., Grünweller A., Ziebuhr J. The rocaglate CR-31-B (−) inhibits SARS-CoV-2 replication at non-cytotoxic, low nanomolar concentrations in vitro and ex vivo. Antiviral Res. 2021; 186:105012. 33422611 10.1016/j.antiviral.2021.105012 PMC7791309

[38] Appelberg S. , Gupta S., Akusjärvi S.S., Ambikan A.T., Mikaeloff F., Saccon E., Végvári Á., Benfeitas R., Sperk M., Ståhlberg M. et al. Dysregulation in Akt/mTOR/HIF-1 signaling identified by proteo-transcriptomics of SARS-CoV-2 infected cells. Emerg. Microbes Infect. 2020; 9:1748–1760. 32691695 10.1080/22221751.2020.1799723 PMC7473213

[39] Terrazzano G. , Rubino V., Palatucci A.T., Giovazzino A., Carriero F., Ruggiero G. An open question: is it rational to inhibit the mTor-dependent pathway as COVID-19 therapy?. Front. Pharmacol. 2020; 11:856. 32574238 10.3389/fphar.2020.00856 PMC7273850

[40] Karam B.S. , Morris R.S., Bramante C.T., Puskarich M., Zolfaghari E.J., Lotfi-Emran S., Ingraham N.E., Charles A., Odde D.J., Tignanelli C.J. mTOR inhibition in COVID-19: a commentary and review of efficacy in RNA viruses. J. Med. Virol. 2021; 93:1843–1846. 33314219 10.1002/jmv.26728 PMC8159020

[41] Mullen P.J. , Garcia G., Purkayastha A., Matulionis N., Schmid E.W., Momcilovic M., Sen C., Langerman J., Ramaiah A., Shackelford D.B. et al. SARS-CoV-2 infection rewires host cell metabolism and is potentially susceptible to mTORC1 inhibition. Nat. Commun. 2021; 12:1876. 33767183 10.1038/s41467-021-22166-4 PMC7994801

[42] Li M.M.H. , MacDonald M.R., Rice C.M. To translate, or not to translate: viral and host mRNA regulation by interferon-stimulated genes. Trends Cell Biol. 2015; 25:320–329. 25748385 10.1016/j.tcb.2015.02.001 PMC4441850

