# Supplementary material for: Cap-independent translation and a precisely located RNA sequence enable SARS-CoV-2 to control host translation and escape anti-viral response
Source: Nucleic Acids Res. 2022 Jul 18;50(14):8080–92. doi: 10.1093/nar/gkac615 (PMC9371909; doi:10.1093/nar/gkac615)
Supplement: gkac615_Supplemental_Files [file gkac615_supplemental_files.zip › SFigs_Slobodin_updated.pdf]

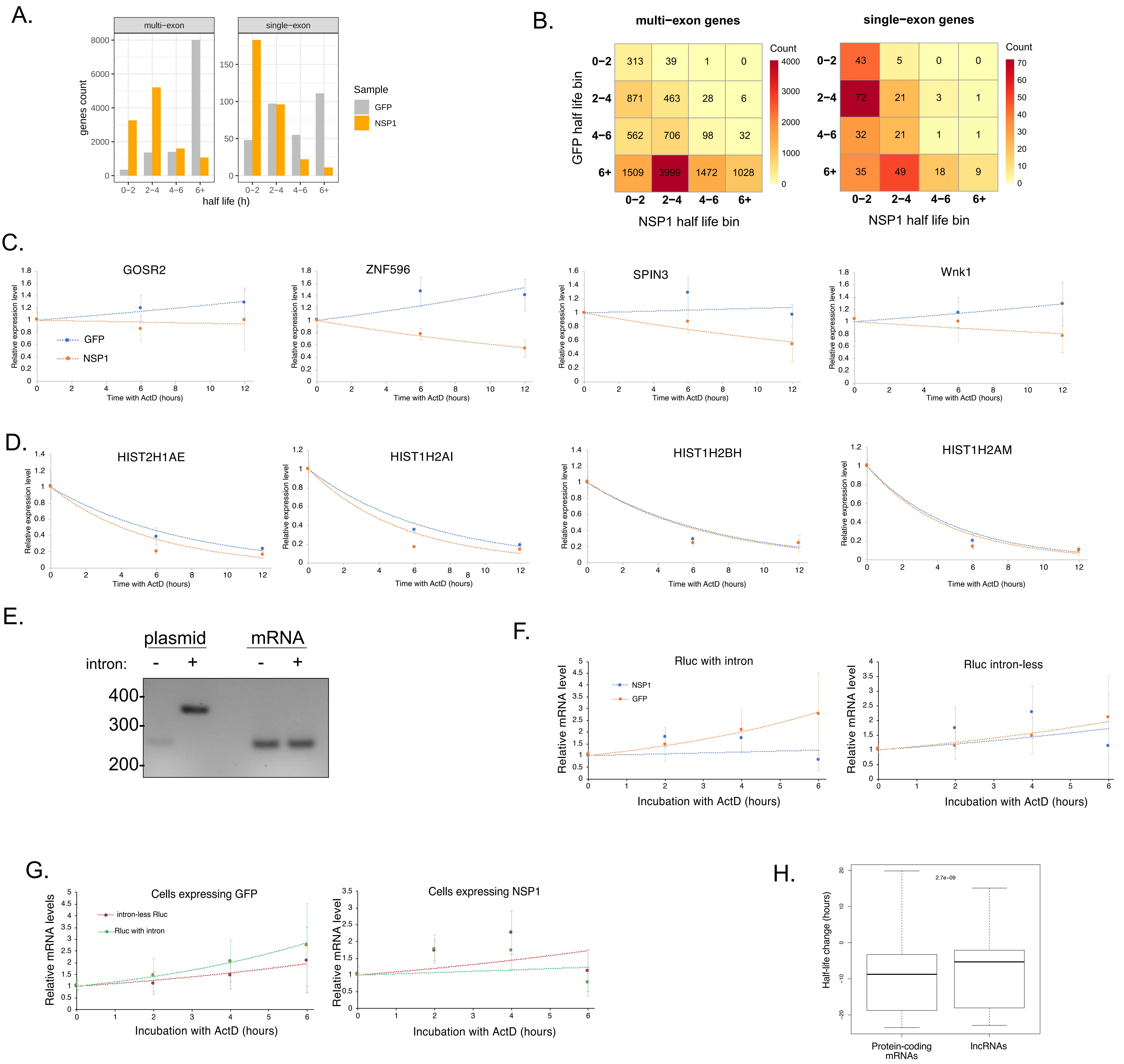

Figure S1: Effects of NSP1 on mRNA stability. A-B: HEK293 cells were transfected and treated with as detailed in Fig. 1A,B, n=2. **A.** Genes were binned according to the half-life of their mRNAs and plotted according to intron/s presence and expression of either HA-NSP1 or eGFP. **B.** Matrix representation of the detected mRNA half-lives. **C-D.** HEK293 cells were transfected with either GFP or HA-NSP1 and 24 hours later treated with Actinomycin D (7.5mg/ml) for 6 and 12 hours, collected and subjected to qRT-PCR analysis; n=3, bars show SE. **C.** Degradation kinetics of mRNAs that showed high stabilities ($T_{1/2}$=24 hours) in the GFP-expressing cells in the MARS-seq experiments (Fig. 1B). Cells were transfected and treated with Actinoycin D as indicated for Fig.1B, collected and subjected to RT-qPCR; n=3, bars represent SE. **D.** Effect of NSP1 on histone mRNAs as representatives of single-exon mRNAs. **E.** Plasmids encoding for Rluc reporter gene with or without intron in its 5'UTR were transfected into HEK293 cells. After collection, both the plasmids and cDNA produced from the isolated RNA were subjected to PCR using primers flanking the intron. **F-G.** HEK293 cells were co-transfected with either GFP or HA-NSP1 along with a mix of plasmids encoding for Rluc reporters with and without intron in its 5'UTR. On the next day, the cells were treated with ActD for 0,2,4 and 6 hours, collected and subjected to qRT-PCR analysis using barcode-targeted specific primers; n=3, bars represent SE. The graphs present the dynamics of mRNA levels according to the presense of intron **(F)** or in NSP1- vs GFP-expressing cells **(G)**. **H.** The effect of NSP1 on the stabilities of protein-coding mRNAs versus polyadenylated long non-coding RNAs in the experiments described in Fig. 1B.

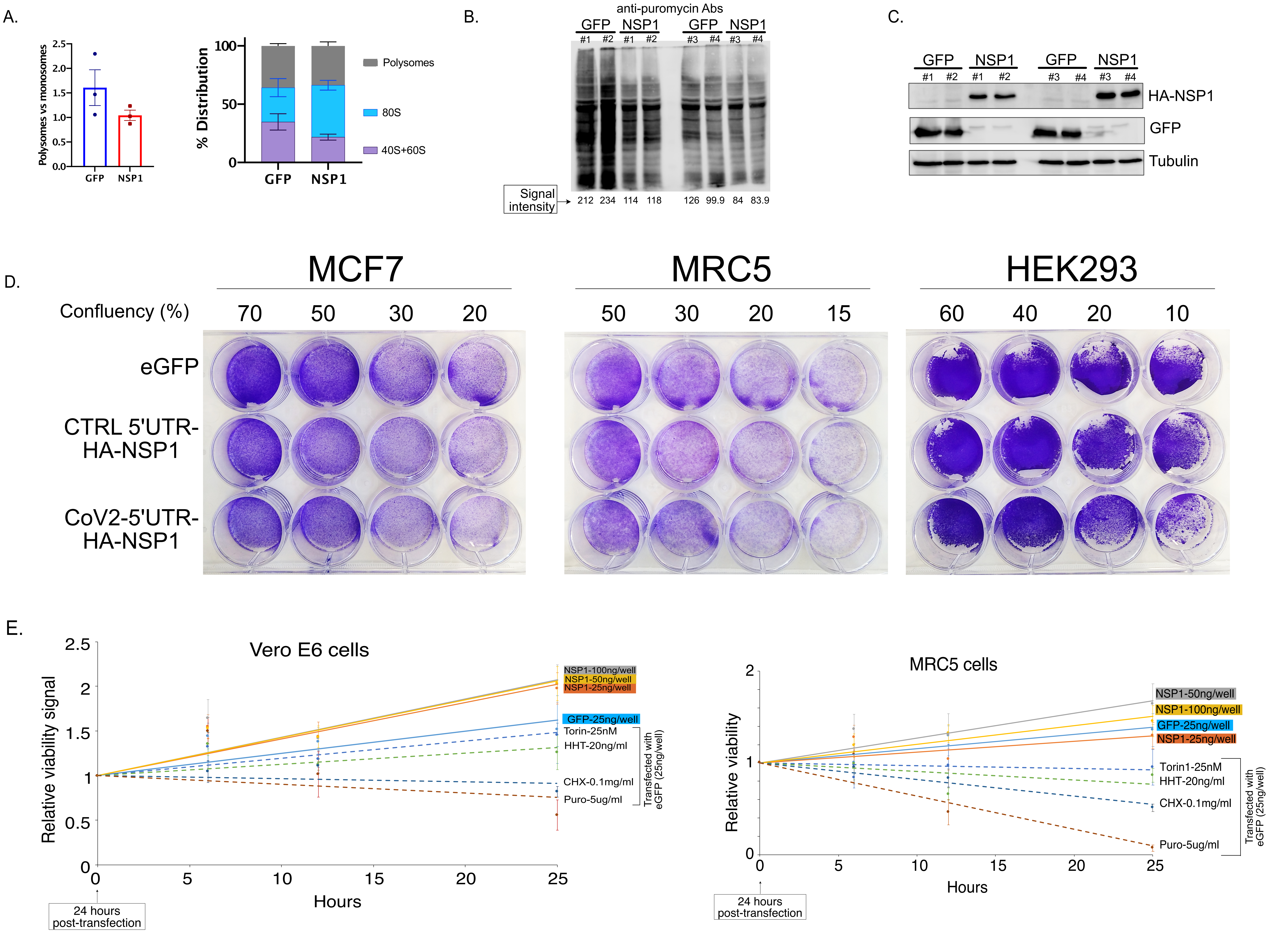

Figure S2: Impact of NSP1 on translation and cell survival. A. Statistical analysis of the polysomal profiling experiments of MCF7 cells as detailed in Fig. 2A. The left graph shows the relative signals in the polysomal vs monosomal fractions. The right graph shows the relative RNA distributions over the different cumulative fractions of the polysomes; n=2, bars show STD. **B.** Original images of the puromycin labeling experiments presented in Fig. 2B showing probing with anti-puromycin antibodies. **C.** Western blot analysis of proteins expression from the experiments presented on Fig. S1B. In both (B) and (C), four experimental repeats are shown. **D.** Indicated cell lines were seeded in serial dilutions into 12-wells plates and on the next day transfected with 200ng of plasmids encoding either eGFP or HA-NSP1 preceded by the different 5'UTRs. The confluencies at the day of transfection are indicated. After 4 hours of transfection, the medium was changed and the cells were grown for additional two days after which they were fixed and stained using crystal violet. **E.** MRC5 and Vero cells were treated as detailed in Fig. 2E and their growth kinetics were detected using quantitative CellTiter-Glow assay at the indicated time points; n=3.

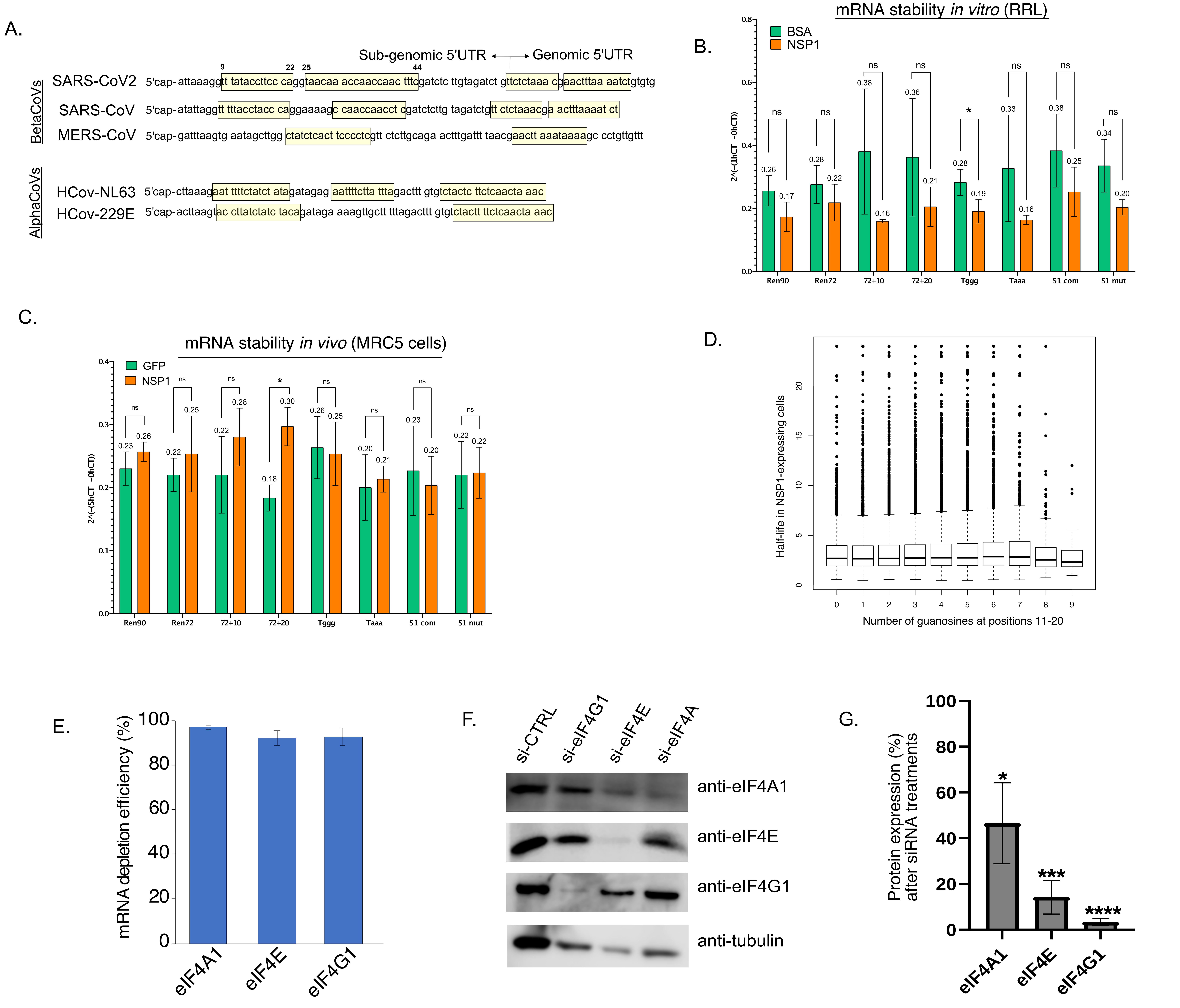

Figure S3. A. Guanosine-deficient stretches (yellow boxes) in the cap-proximal regions of the indicated coronavirus-derived 5'UTRs. **B.** Rabbit reticulocyte lysates (RRL) were pre-incubated with recombinant HA-NSP1 or BSA (5ng/µl) for 10 mins at room temperature and then for 0 or 60 mins with the detailed pre-mixed mRNAs (10ng/µl). After collection, the RNA was extracted, subjected to RT-qPCR with primers targeting the specific barcodes specific for each mRNA; n=3, bars represent SD. **C.** MRC5 cells were transfected with 250ng/35-mm well of plasmid encoding for either eGFP or HA-NSP1. After 24 hours additional transfection of mixed barcoded mRNAs (200 ng/well) was performed and the cells were collected immediately after the transfection or following 5-hour incubation. Isolated RNA was subjected to RT-qPCR using barcode-specific primers; n=3, bars represent SD. **D.** Experiment presented in Fig. 1B was re-analysed to reflect the impact of guanosine residues between nucleotides 11-20 on the mRNA stabilities in the presence of NSP1; n=2. **E-G.** Knock-down efficiencies of the applied siRNAs. **E.** RNA extracted from MRC5 cells transfected with the indicated siRNAs was subjected to RT-qPCR analysis using primers targeting the respective genes and normalized to the expression levels of GAPDH and the respective expression levels in cells expressing non-targeting (control) siRNA. Reduction in mRNA abundance indicates siRNA efficiency; n=3, bars represent SD. **F.** The cells were transfected with the indicated siRNAs, harvested and subjected to Western blot analysis of the indicated proteins; representative image is shown. **G.** Three independent Western blot images were analyzed using ImageStudio software (LI-COR) software, quantifying the expression of the indicated proteins against their respective tubulin levels; n=3.

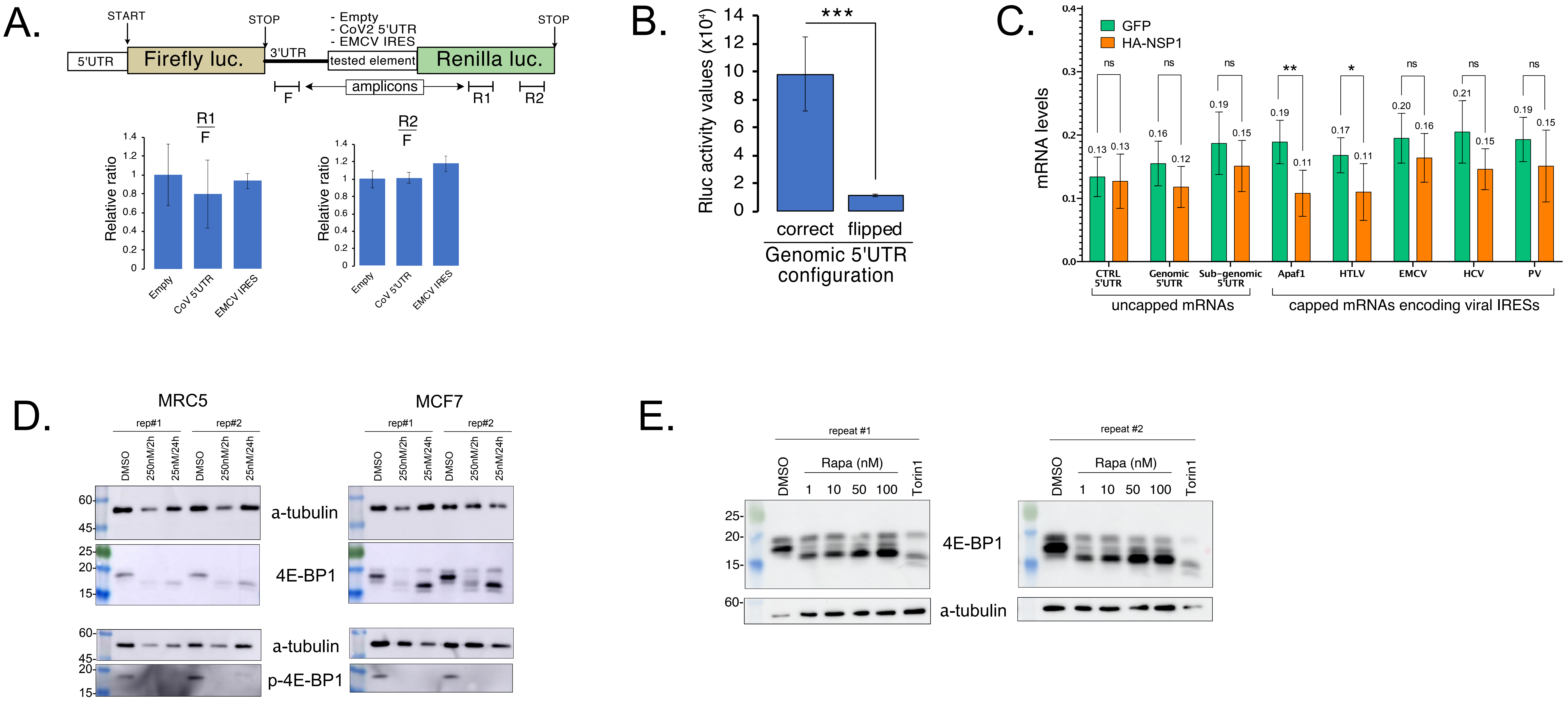

Figure S4. A. MCF7 cells growing in 6-well dishes were transfected with the indicated bi-cistronic plasmids and harvested after 24 hours. After RNA extraction, reverse transcription was performed independently on each RNA lysate using gene-specific primers (i.e., Renilla- and Firefly-specific, #50 & 51 in the Table S1) in separate reactions. Primers targeting the 3'UTR of Firefly and the two regions of Rluc (see schematic amplicons) were used for RT-qPCR; n=3, bars represent SD. **B.** MRC5 cells were transfected with *in vitro* transcribed uncapped Rluc mRNAs preceded by SARS-CoV2-derived 5’UTRs introduced in the correct or "flipped" configuration. Rluc activity was assayed 7 hours after the beginning of transfection; n=4, bars represent SE. **C.** MRC5 cells were transfected with either GFP or HA-NSP1 and in vitro transcribed mRNAs encoding for the detailed UTRs. The cells were collected immediately following or 5 hours after the mRNA transfection. Isolated RNA was subjected to RT-qPCR analysis; n=5, bars represent SD. **D.** MRC5 (left) and MCF7 (right) cells were treated with Torin1 for the indicated concentrations and times. After harvest, 5µg from total protein lysates were resolved on two separate 12% SDS-PAGE and probed with the indicated antibodies; n=2. **E.** MRC5 cells were treated with Rapamycin at the indicated concentrations or with Torin1 (200nM) for 5 hours. After harvesting, 20µg of the total protein lysates were resolved on gels and probed with the indicated antibodies; n=2.
