# Supplementary material for: Cap-independent translation and a precisely located RNA sequence enable SARS-CoV-2 to control host translation and escape anti-viral response
Source: Nucleic Acids Res. 2022 Jul 18;50(14):8080–92. doi: 10.1093/nar/gkac615 (PMC9371909; doi:10.1093/nar/gkac615)
Supplement: gkac615_Supplemental_Files [file gkac615_supplemental_files.zip › Supplementary Tables legends.docx]

## Supplementary Tables

**Table S1** **– Oligonucleotides used in this study.**

**Table S2 – Raw luciferase data.** This table presents raw data collected in the respective luciferase assays; the background signals were subtracted.
